# Intraspecific Trait Variation in Seedlings Reveals Independence Between Leaf and Root Traits but a Lack of an Independent “Collaboration Axis” Belowground

**DOI:** 10.1002/pei3.70019

**Published:** 2024-11-22

**Authors:** Samuel A. Z. Schaffer‐Morrison, Inés Ibáñez, Monique Weemstra, Lais Petri, María Natalia Umaña

**Affiliations:** ^1^ University of Michigan Department of Ecology and Evolutionary Biology Ann Arbor Michigan USA; ^2^ University of Michigan School for Environment and Sustainability Ann Arbor Michigan USA; ^3^ Wageningen University Department of Environmental Sciences Wageningen Netherlands; ^4^ Michigan State University Plant Biology East Lansing Michigan USA

**Keywords:** canopy openness, functional traits, soil nutrients, temperate seedlings, trait covariation

## Abstract

Plant functional traits help determine resource acquisition strategies. Global trends at the interspecific scale suggest independence between leaf and root traits described by three functional dimensions: resource acquisition above‐ and belowground and degree of mycorrhizal collaboration belowground. However, there are ecological and evolutionary reasons to expect different patterns of variation within species, especially within seedlings—the stage at which most tree mortality occurs. Describing the intraspecific patterns of trait variation in seedlings will improve the understanding of tree populations' ability to cope with environmental change. We ask the following questions: (1) How do traits above‐ and belowground co‐vary within species? (2) How do traits relate to soil nutrients and light conditions? We collected root and leaf traits on 131 seedlings from four naturally occurring woody species across eight sites in a temperate, deciduous broadleaf forest in the USA. We measured traits reflecting resource use strategies—specific leaf area, leaf nitrogen, root nitrogen, and root tissue density—and those defining the collaboration axis—specific root length and root diameter. We measured light conditions for each seedling and soil nitrogen and phosphorus to examine the relationship between traits and abiotic conditions using a novel multivariate regression analysis approach. We found that above‐ and belowground traits segregated into independent functional axes and that the collaboration axis merged with the belowground resource‐acquisition axis. We found limited associations between abiotic factors and traits. Our findings suggest that within species, there might be additional constraints to adjust to soil conditions and therefore impact response to environmental change.

## 1 Introduction

Functional traits mediate interactions among organisms and the environment determining individuals' performance. Studies of functional trait variation have been central in the development of mechanistic models that project plant species responses to future climate (Myers‐Smith, Thomas, and Bjorkman 2019; Heilmeier 2019). A key step in building a more mechanistic understating of how plants and ecosystems function is to define the relationship between above‐ and belowground trait variation that shapes functional strategies within species, fostering knowledge on potential adjustments in physiology to changing climate (Meier and Leuschner 2008; Reich 2014; Anderegg 2015; Díaz et al. 2016). Among the well‐studied functional trait dimensions, the leaf economics spectrum (LES) has generated substantial attention. The LES describes functional trait variation that ranges from acquisitive to conservative leaf traits (Wright et al. 2004; Reich 2014). This axis not only describes how plants efficiently acquire and process light but also underscores the tradeoff between rapid carbon acquisition and resource conservation (Wright et al. 2004; Reich 2014). In contrast, belowground traits are thought to be organized in a bidimensional root economics space (Bergmann et al. 2020; Carmona et al. 2021; Weigelt et al. 2021; Beccari and Carmona 2024). One of these root axes resembles the acquisitive–conservative tradeoff seen in the LES, where fine root traits vary along a spectrum with a high nitrogen concentration in roots at the acquisitive end and roots with high root tissue density (RTD; root mass per unit volume) at the conservative end (Bergmann et al. 2020; Weigelt et al. 2021). The second root axis reflects a “collaboration axis” that has been hypothesized to describe the degree to which roots associate with mycorrhizal fungi (Bergmann et al. 2020). One extreme of this collaboration axis is occupied by species that have long, thin roots (i.e., high specific root length (SRL); root length per unit root dry mass), able to efficiently explore the soil and acquire resources. The other extreme is occupied by species that have thick roots (high root diameter (RD)) that cannot explore the soil efficiently by themselves; instead, plants “outsource” the acquisition of soil resources to mycorrhizal fungi, facilitating their colonization with increased RD.

Across species at global scales, these leaf and root traits have been shown to form independent functional dimensions (Medeiros et al. 2017; Carmona et al. 2021; Weigelt et al. 2021; Beccari and Carmona 2024), suggesting some degree of independence between above‐ and belowground resource acquisition functions. However, we may expect different relationships when trends are examined within species, as additional constraints may be at play and limit trait variability, including increased covariation within organs and selection pressure above‐ and belowground from environmental factors (Messier, McGill, and Lechowicz 2010; Messier et al. 2017). For example, trait–trait relationships in the LES tend to weaken or even reverse when intraspecific information is considered, and within grassland plant species, belowground traits tend to co‐vary (Messier, McGill, and Lechowicz 2010; Messier et al. 2017; Anderegg et al. 2018; Umaña and Swenson 2019; Mueller, Kray, and Blumenthal 2024). Examining if functional leaf and root trait axes exhibit similar patterns intra‐ and interspecifically should provide valuable insights into the coordination in above‐ and belowground tree functional strategies, especially as it relates to plant species' ability to cope with changing environments.

Within species, above‐ and belowground traits may still be decoupled due to constraints that operate more strongly among traits within the same organ than across organs (Merila and Bjorklund 2004). This occurs because traits from the same organ tend to participate more cohesively in similar functions and/or share similar organ construction costs (and can be mathematically correlated) (Berg 1960; Poorter and Villar 1997). This shared functionality may result in stronger covariation among belowground traits than between below‐ and aboveground traits and promote covariation between traits that are independent at interspecific scales where these constraints are absent. However, this stronger association among traits from the same organ may weaken the observed dimensionality of the root traits across species, as root traits have shown strong phylogenetic conservatism, even more so than leaf traits (Comas and Eissenstat 2009; Chen et al. 2013; Valverde‐Barrantes et al. 2017). Furthermore, tree species tend to exhibit high specificity with mycorrhizal types (Smith and Read 2008), which can further limit independence in variation across root traits.

In addition, we may expect decoupled variation between leaf and root traits due to the decoupled environmental variation between above‐ and belowground resources, which results in differential patterns of trait variation within a species via plasticity or genetic variation (e.g., local adaptation) (Messier, McGill, and Lechowicz 2010; Messier et al. 2017). For example, at local scales, light heterogeneity is typically associated with tree fall gaps and may influence the distribution of leaf traits; under low light conditions, trees display acquisitive leaves with high nutrient content and a high specific leaf area (SLA; leaf area per unit leaf mass) to maximize carbon metabolism (Wright et al. 2006; Markesteijn, Poorter, and Bongers 2007; Petriţan, von Lüpke, and Petriţan 2008; Barton 2023). Meanwhile, soil nutrients vary at a different spatial scale mainly associated with the distribution of microorganisms, soil heterogeneity, vegetation type, climate, and/or geological processes (Jenny 1994; Vitousek 2004; Fowler et al. 2013). This variation in soil nutrients has been shown to have strong effects on the diversity of belowground traits. When plant available soil nutrient levels are high, roots tend to possess higher nutrient concentrations, while at lower soil nutrient levels, plants may have higher RTD to extend the lifespan of the root and conserve its resources (Eissenstat et al. 2015; Cheng et al. 2016). The decoupled spatial scales at which variation in above‐ and belowground resources occur can thus result in independent variation in above‐ and belowground traits when examined within species (Asefa et al. 2022; Weemstra et al. 2022).

Since multiple traits often respond simultaneously to environmental conditions, it is important to account for the potential coordination (and lack of it) among different traits that participate in similar functions. However, most work relating traits to the environment has done so in a univariate framework (e.g., Freschet et al. 2013; de la Riva et al. 2018; Defrenne et al. 2019), in which each trait–environment relationship is examined independently. This approach ignores the covariation between traits, which is widely accepted to exist and is fundamental for determining functionality, as multiple traits tend to work together to achieve specific functions (e.g., LES and carbon acquisition) (Wright et al. 2004; Armbruster et al. 2014). This integrated functionality is important across organizational levels, especially at the intraspecific scale because, as mentioned above, traits have the potential to be more constrained within species.

This study aims to gain an understanding of the coordination and intraspecific variation of leaf and root traits as well as the extent to which trait variation is explained by light and soil nutrient heterogeneity. We examined four widespread and commonly co‐occurring woody species from forests in the northeastern United States. We focus on seedlings of these species because the seedling stage is essential to the recruitment process in forests, so understanding the functional strategies and environmental responses of seedlings of these species may provide insights into the composition of these forests in the future (Harper 1977; Barnes and Wagner 2004; Green, Harms, and Connell 2014; Lee and Schaffer‐Morrison 2024). The selected species represent different ecological strategies with respect to shade and drought tolerance and form associations with different mycorrhizal types (Table 1). We address the following two questions:

1. How do above‐ and belowground traits within species co‐vary? Owing to differences in the variation of light and soil nutrients both within and between sites, we expect to find weak relationships between comparable traits in roots and leaves (e.g., root N (RtN) and leaf N (LfN)), resulting in independent above‐ and belowground resource‐acquisition axes. We also expect that there will be strong relationships between traits within individual organs driven by additional constraints that ensure optimal organ‐level functionality.
2. How does intraspecific variation in traits relate to abiotic factors, and how does smaller scale (i.e., individual and site level) variation in light and soil nutrients versus variation at larger scales (i.e., climatic differences between northern or southern sites) influence these relationships? Furthermore, is one scale more closely associated with trait variation than the other? Belowground, we expect variation in traits to be associated with variation in N, as N tends to be the most limiting nutrient in these systems (Vitousek 2004; Du et al. 2020). Finally, we predict that seedlings in northern sites that experience a shorter growing season will be selected to maximize growth during that short window and so express a more acquisitive strategy than those in the southern sites with more mild climates (Pavanetto et al. 2024; Midolo 2024). Overall, we expect seedlings to be more associated with smaller scale variations in abiotic variables because smaller variations in the environment can have large impacts on seedlings that lack the same storage capacities and occupy substantially smaller areas than adult trees.

**TABLE 1:** Summary of the life history of the four species used in our study. Mycorrhizal type refers to the primary type of mycorrhizal association, either arbuscular mycorrhizal (AM) or ectomycorrhizal (EcM). Shade and drought tolerance are scored from 0 (no tolerance) to 5 (high tolerance) and include standard deviation. All tolerance data presented is retrieved from Niinemets and Valladares (2006).

Species	Common name	Mycorrshizal type	Shade tolerance	Drought tolerance
*Acer rubrum*	Red Maple	AM	3.44 ± 0.23	1.84 ± 0.16
*Acer saccharum*	Sugar Maple	AM	4.76 ± 0.11	2.25 ± 0.25
*Prunus serotina*	Black Cherry	AM	2.46 ± 0.34	3.02 ± 0.02
*Quercus rubra*	Northern Red Oak	EcM	2.75 ± 0.18	2.88 ± 0.12

## 2 Methods

### 2.1 Study Sites

Our study took place across eight different sites in Michigan, USA (Table 2). Four sites in the southern part of the state (southern sites) (42.2828^o^N, 83.7311^o^W) and four sites in the northern portion of the Lower Peninsula (northern sites) (44.5586^o^N, 84.6778^o^W) were selected. Sites differed in their abiotic environmental variables (Table 2). Available soil nitrogen (N) ranged from 1.06 to 7.13 ppm across all sites, and available soil phosphorus (P) ranged from 0.18 to 3.38 ppm. Average growing season soil moisture ranged from 8% to 30%. Light availability—measured at each seedling location—ranged from 0% to 65% canopy openness. Mean July temperature varied from 15.73°C to 19.47°C, and relative humidity was consistent across all sites (Table 2). N availability and mean July temperature varied significantly between northern and southern sites (*t* = 10.71, *p* < 0.001 and *t* = 13.40; *p* < 0.001 respectively).

**TABLE 2:** Environmental variables for the eight sites used in the study. Location refers to latitude within the lower peninsula of Michigan, USA. All values for soil variables (available nitrogen (N) and phosphorus (P), nitrate, and ammonium) are in ppm. Canopy openness is the mean percent canopy cover across all individuals from all species at each site ± standard deviation (letters indicate differences between sites according to ANOVA). Soil moisture (SM) is an average of the SM measured every hour at roughly the center of each site. Mean temperature in July and relative humidity (RH) were measured at 1 m above the ground level in roughly the center of the site. Southern sites on average had significantly higher total available N, nitrate, and temperature than northern sites according to Welch's *t*‐test.

Location	Available N	Nitrate	Ammonium	Available P	Canopy openness (%)	SM (%)	Temperature (°C)	RH (%)
South1	7.13	3.62	3.51	3.38	8.91 ± 1.21a	10.7 ± 3.7	18.9 ± 3.5	77.64 ± 9.50
South2	5.63	3.74	1.89	0.79	9.53 ± 1.92a	7.8 ± 2.7	19.5 ± 3.4	80.64 ± 11.60
South3	5.84	4.07	1.77	0.76	9.68 ± 1.32a	29.8 ± 5.1	19.5 ± 3.0	80.87 ± 10.22
South4	6.45	4.37	2.07	1.85	9.91 ± 2.13a	30.8 ± 5.0	19.2 ± 2.9	79.49 ± 10.93
North1	1.62	< 0.01	1.62	1.33	10.09 ± 1.04a	16.2 ± 8.2	16.1 ± 3.8	75.08 ± 11.96
North2	1.06	0.00	1.06	0.18	15.76 ± 9.44a	37.6 ± 1.1	15.9 ± 4.6	80.78 ± 15.42
North3	1.71	0.24	1.47	0.33	9.11 ± 1.41a	10.0 ± 6.0	16.6 ± 3.6	78.76 ± 12.02
North4	2.34	0.10	2.25	0.46	23.52 ± 15.32b	18.7 ± 8.4	15.7 ± 5.7	74.83 ± 17.13

### 2.2 Species Selection

We selected four widespread and co‐occurring deciduous tree species commonly found throughout most of the sites included in our study. These species cover a wide range of life‐history strategies (Table 1). *Prunus serotina* Ehrh. (Black Cherry) associates with arbuscular mycorrizal (AM) fungi and is a common pioneer species. *Quercus rubra* L. (Northern Red Oak) is a tree that associates with ectomycorrhizal (EcM) fungi and is moderately shade tolerant throughout its life (Barnes and Wagner 2004). *Acer rubrum* L. (Red Maple) associates with AM fungi and is moderately shade tolerant. *Acer saccharum* Marshall (Sugar Maple) associates with AM fungi and is a highly shade tolerant species.

### 2.3 Sampling Design

In the summer of 2020 at each of the four southern and northern sites, we collected a minimum of three seedlings per species, though at one site, only *Q. rubra* seedlings could be found (see Table S1 for a breakdown of samples taken per site). We define seedlings as individuals that are less than 50 cm in height, are not reproductively mature, and are growing independently (e.g., not an offshoot of a larger tree's root system). Individuals were at least 10 m apart to increase the probability of selecting seedlings from different parent trees.

### 2.4 Functional Traits

In the lab, we measured two leaf traits and four root traits that represent the above‐ and belowground resource acquisition and hypothesized mycorrhizal collaboration strategies for plants (Wright et al. 2004; Bergmann et al. 2020): SLA (cm^2^ g^−1^), LfN (%) content, mean RD (mm), SRL (cm g^−1^), RTD (g cm^−3^), and RtN (%). See the Supporting Information for further details on functional trait collection.

### 2.5 Environmental Data

We compiled data on available soil nitrogen (N, ppm) and phosphorus (P, ppm), and understory canopy openness (%) across all sites. Soil data were measured via resin capsules, and canopy openness was determined via hemispherical photos. All environmental data are presented in Table 2. For more details on the environmental data collection process, see the Supporting Information.

### 2.6 Data Analysis

To identify the coordination between functional traits within species across environments (Question 1), we conducted three different analyses, a principal component analysis (PCA), multifactorial analysis (MFA), and pairwise relationships via Pearson's R between traits within species. We used a PCA to identify axes for above‐ and belowground traits within our four species. To assess the significance of the PCs identified, we used Horn's parallel analysis of principal components (Horn 1965) using the “paran” package in R (Dinno 2018).

Because PCAs are a qualitative way to visually determine independence between trait axes, we used an MFA to quantitatively test independencies between trait axes (i.e., whether traits defining the belowground conservation axis form an independent group from the traits that form the belowground collaboration axis, and whether traits forming the aboveground conservation axis were different from the traits defining the belowground conservation axis) following Baraloto et al. (2010). To perform the MFA, we first ran separate PCAs for (i) the root traits expected to load on the belowground conservation axis (RtN and RTD) and (ii) the root traits expected to load on the collaboration axis (SRL and RD) for each species. We then standardized the original root trait values by dividing them by the square of their own dominant eigenvalues (i.e., highest scores) from their respective PCAs. These standardized values were then used to create a global PCA (i.e., across all individuals within a species) containing traits from both axes, on which we projected the original species‐level trait data. We then calculated the group correlation coefficient (RV) between traits associated with the conservation axis and traits associated with the collaboration axis. The RV ranges from 0 for totally uncorrelated groups of traits to 1 for entirely correlated groups. We compared the observed RV to random RVs generated via 999 permutations of MFAs performed on data created by randomly assigning the original traits to two groups to determine if the traits were more or less correlated than expected by chance (Baraloto et al. 2010). To determine if observed RV values were significantly higher than or less than expected from the null distribution, we employed two‐sided Wilcoxon rank‐sum tests between the observed RV and the distribution of the random RVs. If the observed RV values were significantly lower than the null distribution, this indicates that the trait groups (i.e., trait axes) were independent of one another, and if the observed RV was higher than the null distribution, the trait groups were not independent of one another. The same approach was implemented for testing the independence of the above‐ and belowground traits of the conservation axes. All traits were scaled to mean zero and standard deviation of 1 within a species across all sites before the analysis.

To evaluate how within‐species traits are related to environmental factors that is, light, soil nutrients, and climatic conditions associated with northern and southern sites (Question 2), we implemented a novel modeling approach that accounted for trait covariation within species. To do so, we fitted multivariate/joint response regression models using a Bayesian hierarchical approach via the package “rjags” in R (Plummer 2022). The multivariate response regression model uses a multivariate normal distribution with a mean equal to the trait vector and variance equal to the trait variation–covariation matrix for each species. This multivariate framework constrains the trait–trait variation by factoring in the degree to which each trait varies with each other in determining how the explanatory variables influence each trait. Given the well‐known relationships between traits (e.g., LES), we consider that multivariate/joint response regression models are more appropriate to model the effects of abiotic factors on traits than the univariate models that are typically employed. This is because our multivariate response model considers the degree to which traits co‐vary with one another in a single plant while still providing information on how specific traits relate to the environment (Goldstein et al. 1993; Chung, Fabbri, and Van Westen 1995). While redundancy analysis and the extraction of PC values from trait‐based PCA, as some other papers have done (e.g., Kleyer et al. 2012; Zhang et al. 2018; Midolo 2024), can factor covariation, these methods are unable to describe specific trait‐environment relationships while also being highly sensitive to outliers. The traits included as the response variables were scaled to mean zero and standard deviation one prior to analysis. The explanatory variables were available N, available P, and canopy openness and were standardized and scaled within a species. We also included site random effects to account for differences in climatic conditions in our northern and southern sites, as well as other factors not included in the analysis (e.g., herbivory pressure, soil texture). We fitted a model per species with likelihood as follows:

$${\text{Trait}}_{i}\sim \text{dmnorm}\left(T_{i},S\right).$$

where *Trait*_*i*_ represents a vector of *J* traits from individual *i* of species *s*, and *S* represents the variance–covariance matrix of all traits for species *s*. Each trait *j* of individual *i* from species *s* has the following process model:

$$\begin{array}{c} T_{j,i}={\text{alpha}}_{\text{species}\left(i\right),\text{trait}\left(j\right)}+{\text{beta1}}_{\text{species}\left(i\right),\text{trait}\left(j\right)}\times {\text{Nitrogen}}_{i}\\ +{\text{beta2}}_{\text{species}\left(i\right),\text{trait}\left(j\right)}\times {\text{Phosphorus}}_{i}+{\text{beta3}}_{\text{species}\left(i\right),\text{trait}\left(j\right)}\\ \times {\text{canopy openness}}_{i}+\text{Site}\;{\text{Random Effect}}_{\text{species}\left(i\right),\text{site}\left(j\right)}. \end{array}$$

All parameters (alpha, beta, S, and Site Random Effect [SRE]) were given non‐informative priors with a mean of 0 and a variance of 1000.

To confirm that the covariation included in our multivariate regression models matched the results of the PCA and MFA, we estimated a correlation matrix of traits (ρ) for each species *s* from the variance–covariance matrix of that species (*S*)

$$\rho_{s}=\frac{S_{s}}{{\sqrt{S}}_{s}}$$

All four chains were run for 150,000 iterations, and posterior means were calculated from the final 50,000 iterations with a thinning interval of one. The diagnostic statistic *R̂* = 1.0 was used to check for model convergence for each variable. All model code is provided in the Supporting Information. All analyses were performed using R statistical software (R Core Team 2022).

## 3 Results

### 3.1 Intraspecific Trait Axis Variation and Coordination

**FIGURE 1:**
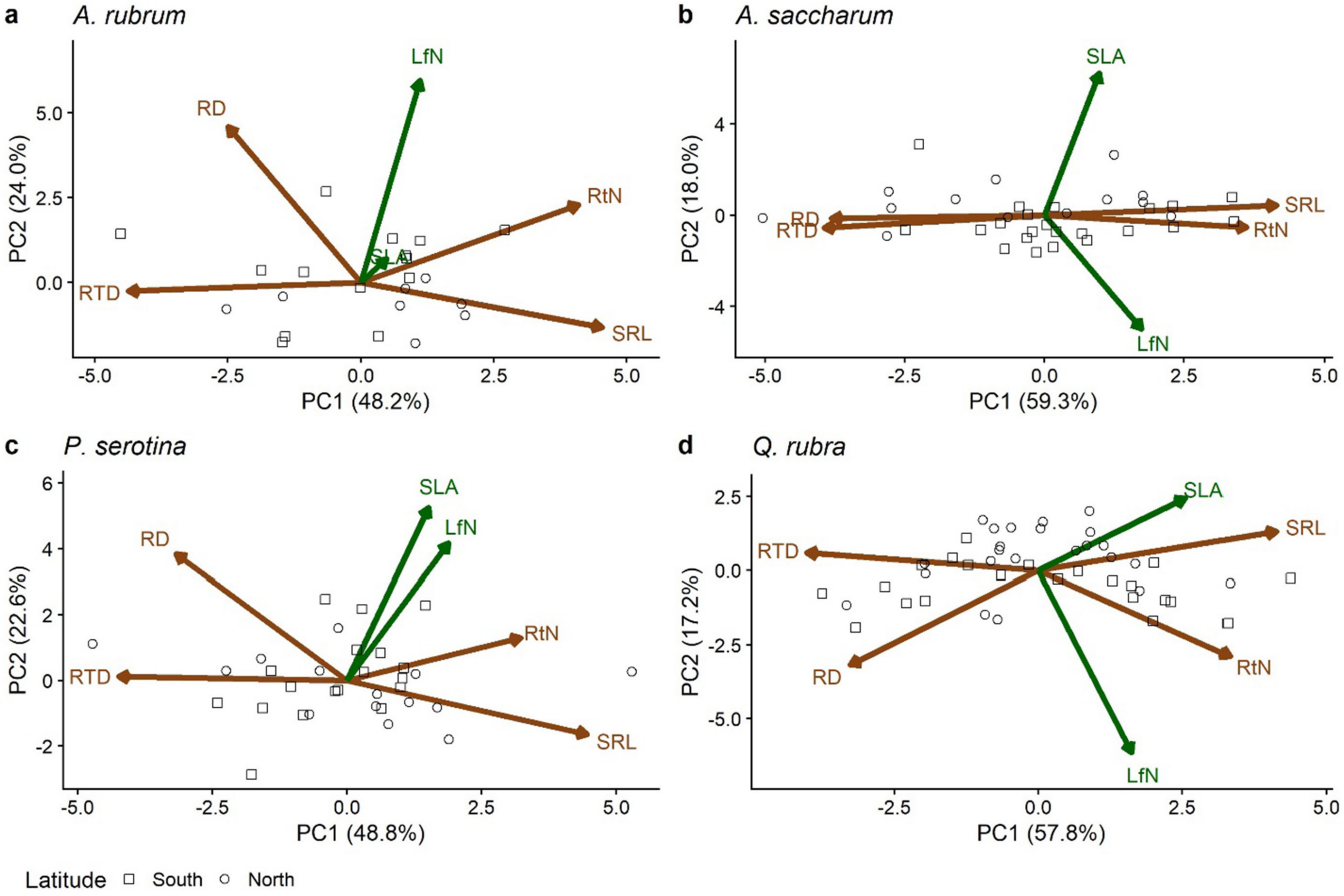
Principal component analysis (PCA) of leaf and root functional traits for *Acer rubrum* (a), *Acer saccharum* (b), *Prunus serotina* (c), and *Quercus rubra* (d). LfN: Leaf nitrogen (ppm), SLA: Specific leaf area (cm^2^ g^−1^), RtN: Root nitrogen (ppm), SRL: Specific root length (cm g^−1^), RTD: Root tissue density (g cm^−3^), and RD: Root diameter (mm). Vectors (traits) are colored according to their association with aboveground (green) or belowground (brown), and point shape indicates location in a northern (circle) or southern site (square). All species show a similar tradeoff between SRL/RtN and RTD/RD on the first PC. The traits associated with the second axis (y) were more variable across all species.

For all species' PCAs, only the first two PC axes were significant (*p* < 0.05). PC1 explained 48.2%, 59.3%, 48.8%, and 57.8% of the variation in the six leaf and root traits for *A. rubrum* , *A. saccharum* , *P. serotina* , and *Q. rubra* , respectively (Figure 1). The first PC was positively associated with SRL and RtN and negatively associated with RD and RTD for all species, and for *Q. rubra*, SLA also was positively loaded on this axis (Figure 1a–d). The second PC explained 24.0%, 18.0%, 22.6%, and 17.2% of the variation in traits for *A. rubrum* , *A. saccharum* , *P. serotina* , and *Q. rubra* , respectively, and was associated with different traits between species (Figure 1a–d). SLA positively loaded on the second axis for all species except for *Q. rubra* (Figure 1a–d). Leaf N was positively loaded on the second axis for *A. rubrum* and *P. serotina* but negatively loaded for *A. saccharum* and *Q. rubra* (Figure 1a–d). All loadings are reported in Table S3.

**FIGURE 2:**
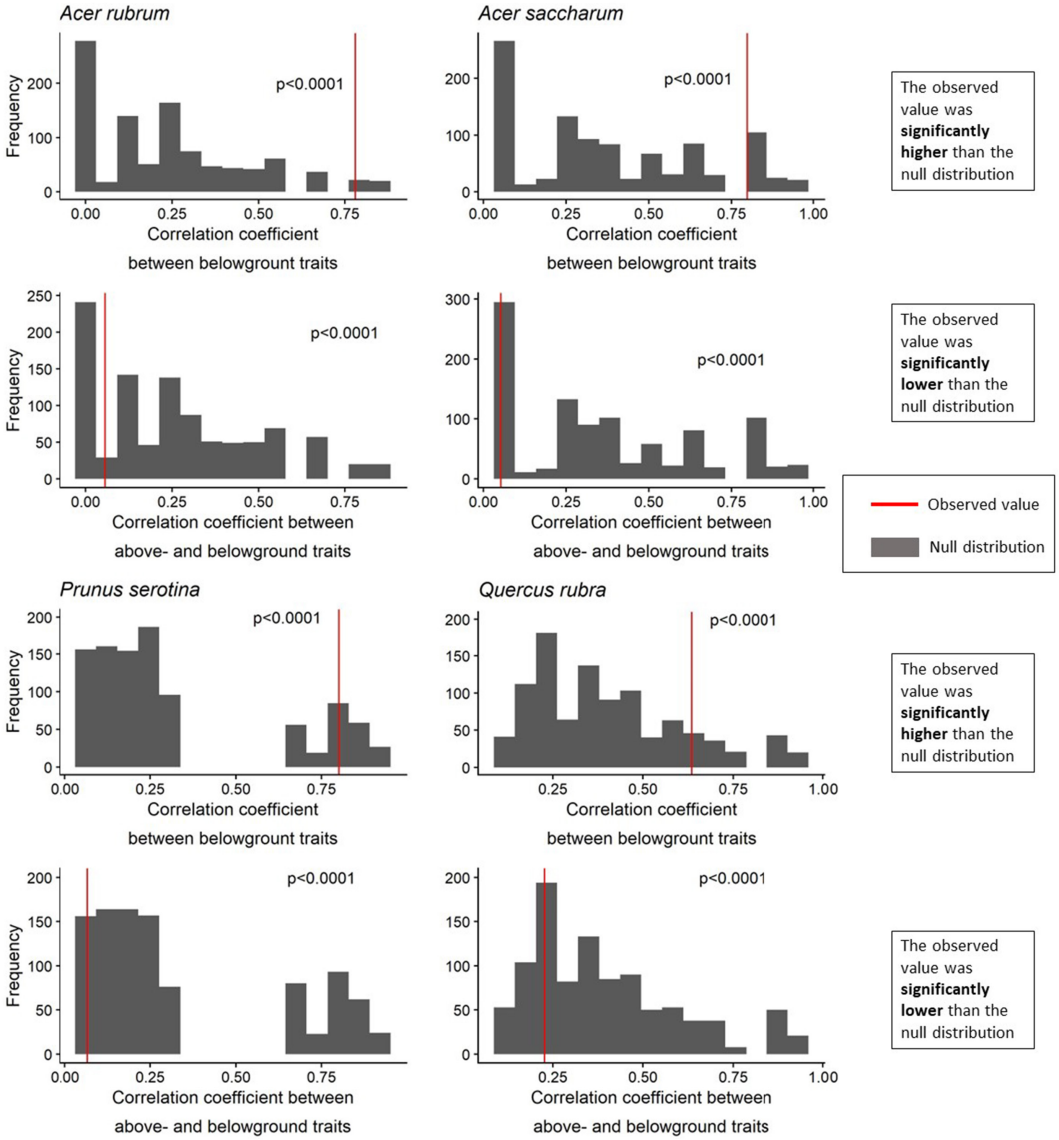
Results of the multifactorial analysis (MFA) between groups of root traits (top row) and between groups of leaf and root traits (bottom row) for (a) *Acer rubrum* , (b) *Acer saccharum* , (c) *Prunus serotina* , and (d) *Quercus rubra* (columns). Axes for plots at the top row are collaboration (SRL/RD) and conservation (RtN/RTD). Axes for plots at the bottom row are aboveground (LfN/SLA) and belowground (RtN/RTD). The red line indicates the observed RV: The group correlation coefficient describes the degree to which the two groups of traits are correlated with one another ranging from 0 (not correlated at all) to 1 (fully correlated). The null RV was generated via 999 permutations of randomly assigned traits to one of the axes. The reported p‐values are calculated via the Wilcoxon test between the generated null distribution RV and the observed RV. A *p* < 0.05 indicates that the observed RV is significantly different than the null RV. When the *p* < 0.05 and the observed RV is shifted to the left on the null distribution, the axes are less coordinated than expected, and when the observed is shifted to the right, the axes are more coordinated than expected. Tabular results are presented in Table S4.

The MFA and subsequent RV comparison supported the pattern that all belowground traits formed a single axis and showed support for the lack of a relationship between above‐ and belowground traits (Figure 2). For all species, the root traits that have been proposed to define the conservation axis (RtN and RTD) and the traits that traditionally define the collaboration axis (SRL and RD) were not independent from each other according to Wilcoxon rank‐sum tests (all *p* < 0.0001; Figure 2 and Table S4). In examining the independence of the above‐ and belowground conservation axes (i.e., LfN and SLA [aboveground] and RtN and RTD [belowground]) the MFA analysis revealed that traits associated with the conservation‐acquisition axes for above‐ and belowground were significantly independent from one another according to Wilcoxon rank‐sum tests (all *p* < 0.0001; Figure 2 and Table S4).

After accounting for environmental context and covariation among traits, the trait–trait relationships via pairwise correlations estimated by the Bayesian analysis partially supported the PCA and MFA results (Table S5). RD and SRL were strongly negatively correlated. Root traits typically associated with the belowground conservation axis (RtN and RTD) were negatively related within all species, but this correlation was only significant for *A. saccharum* . Like in the PCA and MFA, we found significant correlations between all root traits, suggesting a single belowground axis rather than two independent collaboration and conservation axes. For example, RD and RTD were significantly positively correlated with each other for all species except for *A. rubrum* . Likewise, RtN and SRL were significantly positively associated with each other in all species except for *P. serotina* . Overall, some combinations of RD and RTD were significantly negatively correlated with RtN and SRL across all species, supporting the belowground tradeoff observed in the PCAs. Aboveground, while the PCAs indicated a positive relationship between LfN and SLA, supporting the LES for *A. rubrum* and *P. serotina* , and a negative relationship for *A. saccharum* and *Q. rubra* , the correlation analysis found no significant relationship for any species between the two traits for any species. *A. saccharum* showed significant negative relationships between LfN and all root traits except for RtN. In general, these results indicate that LES traits and belowground conservation traits were coordinated with one another in *A. rubrum* and *Q. rubra* , and independent of one another in *A. saccharum* and *P. serotina* . Also, the collaboration axis was not entirely independent of the belowground conservation axis for any species. Raw trait data means and variation can be found in Table S2 and Figures S1 and S2.

### 3.2 Trait–Environment Relationships Within Species

**FIGURE 3:**
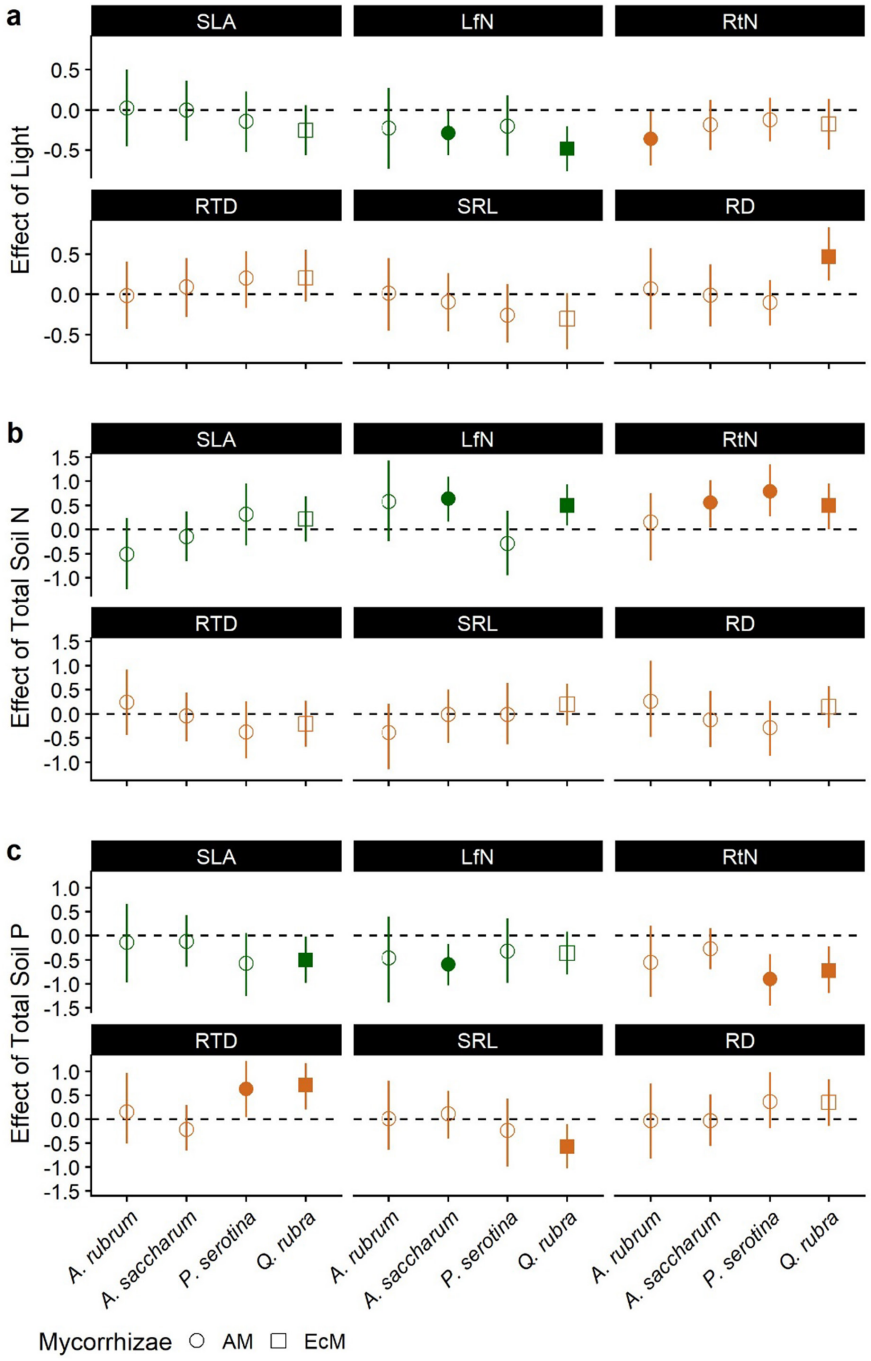
Posterior means of Bayesian hierarchical analysis for the effect of light (a), total available N (b), and total available P (c) for each species and trait combination. Lines represent the 95% credible interval (CI). Symbol shape denotes mycorrhizal type: Circle—arbuscular mycorrhizal (AM) and square—ectomycorrhizal (EcM). When the 95% CI does not cross the zero line, the effect is significant, denoted by a filled shape. Aboveground traits are colored in green, and belowground traits in brown. Trait codes are the same as in Figure 1. Tabular results are shown in the Supporting Information (Tables S6–S12).

Canopy openness had a negative association with LfN for *A. saccharum* and *Q. rubra* , a negative association with RtN in *A. rubrum* , and a positive association with RD for *Q. rubra* (Figure 3a). No other significant relationships between light and traits were found across species (Tables S6–S12).

For soil variables, available soil N in general had positive associations with LfN and RtN. For *Q. rubra* and *A. saccharum* , both LfN and RtN were positively associated with N, while only RtN in *P. serotina* was positively associated (Figure 3b). Available soil P had a negative association with SLA in *Q. rubra* and LfN in *A. saccharum* . It was also negatively associated with RtN for *Q. rubra* and *P. serotina* (Figure 3c). Additionally, the SRL of *Q. rubra* seedlings decreased with increasing levels of soil P, while the RTD of *P. serotina* and *Q. rubra* increased with increased soil P (Figure 3c). Traits did not differ significantly in their associations between northern and southern sites within species. Site random effects do not show any climatic (i.e., site or latitude) differences in any of the species (Table S12).

## 4 Discussion

Characterizing the dimensions of whole‐plant phenotypic variation within species provides a mechanistic basis to understand how plants interact with and respond to their local environment (Messier, McGill, and Lechowicz 2010). Our findings demonstrated that, as previously observed across species, within species, leaf and root traits remain separated along different functional dimensions. However, we showed that root traits, which were previously identified in global studies as forming two independent dimensions (Bergmann et al. 2020; Carmona et al. 2021; Weigelt et al. 2021; Beccari and Carmona 2024), have collapsed into a single root dimension that describes a conservative‐acquisitive axis. Although there was considerable intraspecific trait variation driving changes in trait–trait relationships, our results generally indicate that both leaf and root traits have a weak relationship with the measured abiotic factors; however, smaller scale (i.e., individual and site level) environmental factors were stronger predictors than large scale, climatic differences.

### 4.1 Root Traits Segregated in a Single Multivariate Axis

Intraspecific variation in root traits was aligned along a single dimension, with individuals characterized by low RTD, RD, and high specific root length (SRL), and root N (RtN) on one end, and those with opposite traits on the other end (Figures 1a–d and 2). This result differs from patterns detected at interspecific levels comprising two independent axes of root traits (Bergmann et al. 2020) but corresponds to the hypothesized analogue of the LES for root systems (Mommer and Weemstra 2012; Roumet et al. 2016). The lack of two independent root axes within species might be explained by stronger constraints in trait variation within organs that lead to trait integration (Smith et al. 1985; Schlichting 1989; Armbruster et al. 1999; McGuigan 2006). These constraints may be related to the construction costs and metabolic activity associated with root traits (Penning de Vries, Brunsting, and van Laars 1974; Smith et al. 1985; Laureano et al. 2013). It has been found that across species, chemical (RtN) and morphological (such as SRL, RTD, and RD) traits are significantly related to the fine‐root respiration rate, which represents metabolic activity (Withington et al. 2006; Roumet et al. 2016; de la Riva et al. 2021; Hou et al. 2024). Within species, these metabolic constraints and carbon construction costs may more strongly drive patterns of root trait covariation, resulting in the observed single root trait axis, while across species, additional anatomical and structural differences at the tissue and cellular level may allow for the segregation of root traits in two independent axes. For example, species of graminoids and eudicots exhibit a marked differentiation in RD (Roumet et al. 2016) potentially related to the development of secondary growth in eudicots but not monocots that is a driving force behind anatomical and structural differences in the root xylem (Hummel et al. 2007; Vogel 2008). These types of biophysical differences are unlikely to occur within species.

Furthermore, the lack of independence between the SRL–RD axis, which is hypothesized to represents the collaboration axis in global studies, and the RTD–RtN axis raises questions about general support for the collaboration with mycorrhizal fungi within species. The SRL–RD axis has been proposed to indicate variation in the degree of mycorrhizal association with root traits that offer an alternative range of belowground resource uptake pathways in addition to the conservative‐acquisitive axis (Bergmann et al. 2020). However, our findings suggest that within species, this alternative range of resource uptake strategies tends to disappear. This could be attributed to the limitation in the types of mycorrhizal associations that a particular species develops, and such specialization may constrain the root morphology of the roots to form an independent axis (Brundrett, Murase, and Kendrick 1990; Smith and Read 2008). For example, three of the species in our study develop AM associations, which have limited ability to access soil N (Hawkins, Johansen, and George 2000; Smith and Read 2008). Given the N limitation of temperate forest soils (LeBauer and Treseder 2008), it is possible that these seedlings may rely less on their mycorrhizal symbionts (compared to other AM plants globally of various ontogenetic stages) to explore greater volumes of soil using their own root systems, making all root traits align in a single functional dimension. This may be driven by the fact that seedlings may opt for less reliance on mycorrhizal fungi as the carbon cost of the symbiosis may be too high compared to investment in their own roots, especially in the light‐limited understory (Smith and Read 2008; Ibáñez and McCarthy‐Neumann 2016).

### 4.2 Leaf Economics Traits Tend to be Decoupled From Root Traits Within Species

Leaf and root traits were segregated into independent trait axes and showed weak relationships (Figures 1a–d and 2 and Table S4), indicating that the variation in root traits is not strongly correlated with variation in leaf economics traits and vice‐versa. Similar results have been previously reported across species (Medeiros et al. 2017; Bergmann et al. 2020; Carmona et al. 2021; Weigelt et al. 2021), and in two recent studies, within species (Asefa et al. 2022; Weemstra et al. 2022), suggesting that a decoupled variation between above and belowground traits seems to be common and perhaps a generalizable pattern across and within plant species. These decoupled functional dimensions have implications for the ability of a species to adapt to new and/or shifting environmental conditions. The existence of multiple independent trait axes provides a species the ability to shift the functionality of various organs as necessary to different environmental conditions, increasing the breadth of environments a species can survive in (Baraloto et al. 2010). Moreover, much prior work linking environmental shifts and traits has focused primarily on aboveground traits only (e.g., Wright et al. 2005; Ordoñez et al. 2009); our results here corroborate previous findings (Asefa et al. 2022; Weemstra et al. 2022) and suggest that future work should make sure to include both above‐ and belowground traits to gain better insights into the functional response to the stresses induced by environmental change, which rarely affect isolated environmental variables.

### 4.3 Light, Available N, and Available P are Weak Predictors of Leaf and Root Economics Traits Within Species

In general, traits were more related to smaller scale (i.e., individual and site level) than to larger regional scale (i.e., northern vs. southern Michigan) variation in abiotic factors, as shown by a lack of effect of site location in our join regression model. However, even within sites, traits were only weakly related to abiotic factors (Figure 3a–d). The weak association between light and aboveground traits was not expected. We suggest that these results may be attributed to the fact that all seedlings studied were found in the understory where canopy openness was highly reduced: for 117 out of 131 seedlings (89.31%), light availability was lower than 15%, so there was not a large enough variation in light to detect a relationship with leaf traits. Also, while it is expected that light would have limited association with root traits, we found instances where light was significantly negatively related to RtN in *A. rubrum* and RD in *Q. rubra* (Figure 3a). Prior work has found similar belowground responses to light, typically showing that even at low light levels, an increase in light pushes roots towards a more conservative strategy (lower RtN and/or SRL and higher RD and/or RTD) as we saw in our study (Markesteijn and Poorter 2009; Boonman et al. 2020). This shift toward conservatism is potentially caused by an additional need for water due to elevated light and subsequent investment in deeper, thicker roots to access and transport more water.

The relationships between available soil N and traits matched our expectations and previous results, with leaf and root tissue concentrations of N increasing under higher levels of N (Figure 3b) (Ordoñez et al. 2009; Holdaway et al. 2011; Defrenne et al. 2019; Delpiano et al. 2020). Elevated N in the soil provides the N necessary to increase the amount of machinery for photosynthetic and metabolic activities in the leaves and roots, respectively (Barnes et al. 1997). Interestingly, available soil P was most frequently related to variation in traits across species (Figure 3c). Specifically, *Q. rubra—*the only EcM species in our study—had all traits except for LfN and RD associated with available P. This runs counter to the traditional view that plants should respond more strongly to N than P availability because newer ecosystems such as temperate forests should be more N limited (Vitousek 2004). However, prior work on temperate forests has shown that P can be as or more limiting to plant growth than N (Goswami et al. 2017). Plants tended to opt for a less acquisitive strategy as P levels increased, potentially because fewer resources were needed to search for and acquire P.

While we did find some associations between soil nutrients (N and P) and leaf and root traits, they were not as pervasive as prior studies have shown (Figure 3) (Holdaway et al. 2011; Defrenne et al. 2019). One possible explanation is that the spatial scale at which the variation in P and N was measured does not capture a finer scale variation that is relevant for plants. For example, P is a highly immobile resource that typically has a very patchy distribution in soil, and as a result, there may be areas in a site with substantially higher P concentrations than others (Vitousek et al. 2010). Previous studies have shown that roots respond strongly to this micro‐environmental variability, with root proliferation and traits changing based upon proximity to nutrient patches (Eissenstat et al. 2015; Cheng et al. 2016; Chen, Koide, and Eissenstat 2018).

The general lack of relationships between traits and the environment we found in this study is not necessarily a novel issue, in fact one of the major challenges of functional ecology is poor relationships between traits and the environment (Anderegg 2023). At the intraspecific scale examined here, this may be driven by not only limited variation in environment coupled with high trait variation as mentioned above but also non‐adaptive trait variation. The effects of non‐adaptive trait variation are maximized when examining intraspecific variation in traits because local scale stress may drive phenotypic responses that fail to maximize fitness (Ackerly et al. 2002; Anderegg 2023; Puglielli et al. 2024). This non‐adaptive plasticity can then decouple traits from their environment; in the case of this study, the low light levels may be causing non‐adaptive variation to measured environmental variables.

## 5 Conclusion

In conclusion, our study provides evidence that intraspecific variation in leaf and root traits does not always align with the patterns of variation observed across species. In particular, root traits tend to form a single axis of variation in contrast to the two root axes found across species. This raises questions about the functionality of the collaboration axis within species, and answering these questions requires quantitative data on mycorrhizal colonization rates, associated root traits, and trees of different ontogenetic stages. Finally, our results provide further evidence of the importance of quantifying and exploring intraspecific patterns of variation, as they can differ greatly from patterns seen at larger, interspecific scales.

## Conflicts of Interest

The authors declare no conflicts of interest.

## Supporting information


Data S1.

## Data Availability

Data can be found at https://figshare.com/account/home#/projects/170706.
