# Supplementary material for: Intraspecific Trait Variation in Seedlings Reveals Independence Between Leaf and Root Traits but a Lack of an Independent “Collaboration Axis” Belowground
Source: Plant Environ Interact. 2024 Nov 22;5(6):e70019. doi: 10.1002/pei3.70019 (PMC11584351; doi:10.1002/pei3.70019)
Supplement: Supplementary file 1 — Data S1. [file PEI3-5-e70019-s001.docx]

**Intraspecific trait variation in seedling reveals independence between leaf and root traits but a lack of an independent "collaboration axis" belowground.**

**Supplementary Materials**

**Content:**

- **Supplementary Methods**
- **Table S1. Number of samples per site**
- **Table S2. Species measured trait values**
- **Table S3. PCA loadings**
- **Table S4. Tabular MFA results**
- **Table S5. Correlation matrices from models**
- **Tables S6-S12. Tabular Bayesian model results**
- **Figure S1: Boxplot of Raw Trait Data for Aboveground Traits**
- **Figure S2: Boxplot of Raw Trait Data for Belowground Traits**
- **Model Code**

**Supplementary Methods**

*Seedling Collection*

We excavated seedlings by loosening the soil around each seedling using a shovel and then carefully removing the seedling with the surrounding soil to ensure most fine roots were collected. Seedlings were stored in coolers and returned to the lab, where they were stored at 4^o^C until they were processed (maximum 24 hours for aboveground traits and 10 days for belowground traits).

*Plant Traits*

For leaf traits, we selected one to three fully developed leaves per seedling depending on the number and condition of the leaves present. We dried all leaves at 70ºC for 48 hours before determining their dry mass. We calculated specific leaf area (SLA, cm^2^ g~~ram~~^-1^) by dividing the mean leaf area measured using an LI-3000C portable leaf area meter (LI-COR Inc., Lincoln, NE, USA) by the dry mass of the sampled leaves. Dried leaf samples were analyzed for leaf N (LfN) content.

We cleaned the root system of each seedling using tap water. The fine roots were handled and cleaned very gently to avoid damaging the smallest of the roots. Any roots not connected to the seedling were discarded to eliminate the inclusion of roots from other individuals. Coarse roots – roots over 2 mm in diameter and/or 4^th^ order or higher – were not included in this study.

We scanned fine roots using a flat-bed scanner (Regent STD 4800, Regent Instruments Inc, Canada) at 600 dpi in de-ionized water. Following scanning, the ~~fine~~ roots were dried for ~~at least~~ 72 hours at 70ºC and then weighed to calculate fine root dry mass. The scanned images were analyzed using WhinRhizo 2019 (Regent Instruments Inc, Canada) to determine total root length, the mean diameter of the roots (RD, mm), and total root volume. Per individual, we calculated specific root length (SRL, cm g^-1^) by dividing total length by fine-root dry mass, and root tissue density (RTD, g cm^-3^) by dividing total root volume by fine-root dry mass. Dry root samples were analyzed for root N content (RtN).

*Environmental Data*

from samples that were collected in 2019. For each site, two resin capsules (Unibest Ion Exchange Technologies, Washington, USA) were buried at 10cm depth in the field in May of 2019 and collected in October fall 2019. The resin capsules were analyzed for available N (N, ppm) and phosphorus (P, ppm) content at the University of Michigan Biological Station Analytics Lab.

As a proxy for light availability, we took hemispherical photographs using a fisheye lens (Aukey 180^0^ Fisheye Lens) attached to a smartphone placed above each seedling at a height of 50cm above the soil immediately after seedling collection in 2020. Photographs were converted to black and white in Fiji (Schindelin et al. 2012), with care taken to ensure that no other objects besides canopy coverage were included. Canopy openness (%) was calculated from the black and white photos based on the protocol developed by Bianchi et al. (2017) in R (Bianchi et al. 2017, R Core Team 2022). Temperature and relative humidity were measured continuously using HOBO remote monitoring stations (Onset, Massachusetts, USA), and reported as mean July temperature and mean growing season relative humidity. We determined differences between sites as well as locations (*i.e.,* northern or southern Michigan) using an ANOVA and post-hoc Tukey’s test and Welch’s t-test respectively. All environmental descriptive information and results are presented in Table 2. Light availability was significantly higher in site North4 than all other sites in both northern and southern areas (ANOVA: F = 12.91; p < 0.001).

While soil moisture was measured, it was measured only at a single location within each site. Prior work that our research team has performed at these sites in which we have measured soil moisture shows very high levels of both spatial and temporal variation in soil moisture, so we chose not to include the data here.

**Supplementary Table S1** – Number of samples per site per species included in the study.

| **Species** | **Site** | **Location** | **No. Samples** |
| --- | --- | --- | --- |
| *Acer rubrum* | South 1 | ESGR | 5 |
|  | South 2 | RF | 1 |
|  | South 3 | SF | 6 |
|  | South 4 | SW | 2 |
|  | North 1 | A | 3 |
|  | North 2 | BF | 0 |
|  | North 3 | NH | 1 |
|  | North 4 | PA | 4 |
| *Acer saccharum* | South 1 | ESGR | 5 |
|  | South 2 | RF | 5 |
|  | South 3 | SF | 6 |
|  | South 4 | SW | 6 |
|  | North 1 | A | 5 |
|  | North 2 | BF | 0 |
|  | North 3 | NH | 8 |
|  | North 4 | PA | 0 |
| *Prunus Serotina* | South 1 | ESGR | 5 |
|  | South 2 | RF | 6 |
|  | South 3 | SF | 4 |
|  | South 4 | SW | 3 |
|  | North 1 | A | 3 |
|  | North 2 | BF | 0 |
|  | North 3 | NH | 5 |
|  | North 4 | PA | 10 |
| *Quercus rubra* | South 1 | ESGR | 5 |
|  | South 2 | RF | 7 |
|  | South 3 | SF | 4 |
|  | South 4 | SW | 6 |
|  | North 1 | A | 6 |
|  | North 2 | BF | 8 |
|  | North 3 | NH | 4 |
|  | North 4 | PA | 5 |

**Supplementary Table S2** – Species mean and (standard deviation) for traits. Letters indicate significant differences between species within a trait according to two-way ANOVA. Some but not all traits were overall different across species (Supplementary Table S2). Leaf N was significantly higher for *Q. rubra* than for the other species, and higher for *P. serotina* than *A. saccharum* (ANOVA: df = 3, F = 13.20, p<0.001). Specific root length was significantly higher for *A. rubrum* than *Q. rubra* (ANOVA: df = 3, F = 4.21, p<0.05). *Q. rubra* had the highest RTD, followed by *A. saccharum* (ANOVA: df = 3, F = 8.90, p<0.001). Root N was highest in *P. serotina,* followed by the two *Acer* species, while *Q. rubra* was the lowest (ANOVA: df = 3, F = 16.17, p<0.001).

Specific leaf area (ANOVA: df = 3, F = 2.965, p > 0.05) and RD (ANOVA: df = 3, F = 0.865, p > 0.45) did not differ significantly across species.

| **Species** | **Leaf N (ppm)** | **Specific Leaf Area (g/cm^2^)** | **Specific Root Length (cm/g)** | **Root Diameter (mm)** | **Root Tissue Density (g/cm^3^)** | **Root N (ppm)** | **Canopy Openness (% Cover)** |
| --- | --- | --- | --- | --- | --- | --- | --- |
| *Acer rubrum* | 1.55(0.58)a,b | 178.77(82.84)a | 38.79(11.09)a | 0.38(0.02)a | 0.24(0.07)a | 1.08(0.29)a | 11.78(9.2) |
| *Acer saccharum* | 1.35(0.21)a | 180.98(75.49)a | 31.63(11.21)a,b | 0.38(0.03)a | 0.31(0.07)b | 1.03(0.25)a | 9.65(1.3) |
| *Prunus serotina* | 1.57(0.29)b | 141.15(60.78)a | 36.61(34.68)a,b | 0.4(0.04)a | 0.29(0.09)a | 1.38(0.38)b | 13.0(9.4) |
| *Quercus rubra* | 1.79(0.27)c | 154.76(84.56)a | 27.74(14.80)b | 0.39(0.05)a | 0.36(0.1)c | 0.87(0.29)c | 14.0(11.8) |

**Supplementary Table S3** – PCA loadings for each trait for each species (*Acer rubrum*, *Acer saccharum*, *Prunus serotina*, and *Quercus rubra*). SLA – specific leaf area (cm^2^ g^-1^), SRL – specific root length (cm g^-1^), RD – root diameter (mm), RTD – root tissue density (g cm^-3^), LfN – leaf nitrogen (ppm), RtN – root nitrogen (ppm).

| **Species** | **Trait** | **PC1** | **PC2** | |
| --- | --- | --- | --- | --- |
| *A. rubrum* | SLA | 0.0869 | 0.08690 | |
| *A. rubrum* | SRL | 0.5668 | -0.16592 | |
| *A. rubrum* | RD | -0.312 | 0.5731 | |
| *A. rubrum* | RTD | -0.5455 | -0.0327 | |
| *A. rubrum* | LfN | 0.1392 | 0.7439 | |
| *A. rubrum* | RtN | 0.5107 | 0.2863 | |
| *A. saccharum* | SLA | 0.1227 | 0.7750 | |
| *A. saccharum* | SRL | 0.5167 | 0.0527 | |
| *A. saccharum* | RD | -0.4758 | -0.0185 | |
| *A. saccharum* | RTD | -0.4921 | -0.0703 | |
| *A. saccharum* | LfN | 0.2173 | -0.6219 | |
| *A. saccharum* | RtN | 0.4496 | -0.0680 | |
| *P. serotina* | SLA | 0.1893 | 0.6562 | |
| *P. serotina* | SRL | 0.5564 | -0.2060 | |
| *P. serotina* | RD | -0.3958 | 0.4795 | |
| *P. serotina* | RTD | -0.5294 | 0.0147 | |
| *P. serotina* | LfN | 0.2355 | 0.5200 | |
| *P. serotina* | RtN | 0.4027 | 0.1625 | |
| *Q. rubra* | SLA | 0.3187 | 0.3047 | |
| *Q. rubra* | SRL | 0.5152 | 0.1633 | |
| *Q. rubra* | RD | -0.4080 | -0.3963 | |
| *Q. rubra* | RTD | -0.5027 | 0.0751 | |
| *Q. rubra* | LfN | 0.2029 | -0.7669 | |
| *Q. rubra* | RtN | 0.4154 | -0.3600 | |
|  |  |  |  |  |

**Supplementary Table S5** - Results of the multifactorial analysis (MFA) between groups of leaf and root traits. Axes: Belowground Collaboration (SRL/RD) and Conservation (RtN/RTD); Aboveground (LfN/SLA) and Belowground (RtN/RTD). Observed RV: the group correlation coefficient describes the degree to which two groups of traits are correlated with one another ranging from 0 (not correlated at all) to 1 (fully correlated). Mean and SD null RV were generated via 999 permutations of randomly assigning traits to groups. The reported p-value is calculated via Wilcoxon test between the null distribution and the observed RV. A p-value less than 0.05 indicates that the observed RV is significantly different than the null RV, indicating when the observed is lower that the axes are less coordinated than expected and when the observed is higher the axes are more coordinated than expected.

| **Species** | **Axes** | | **Observed RV** | **Mean±SD null RV** | | | **p-value** |
| --- | --- | --- | --- | --- | --- | --- | --- |
| *A. rubrum* | Belowground Collaboration - Conservation | 0.780 | | | 0.251**±**0.226 | <0.0001 | |
| *A. rubrum* | Aboveground - Belowground | 0.057 | | | 0.251**±**0.236 | <0.0001 | |
| *A. saccharum* | Belowground Collaboration - Conservation | 0.798 | | | 0.360**±**0.271 | <0.0001 | |
| *A. saccharum* | Aboveground - Belowground | 0.053 | | | 0.363**±**0.274 | <0.0001 | |
| *P. serotina* | Belowground Collaboration - Conservation | 0.800 | | | 0.327**±**0.282 | <0.0001 | |
| *P. serotina* | Aboveground - Belowground | 0.066 | | | 0.342**±**0.282 | <0.0001 | |
| *Q. rubra* | Belowground Collaboration - Conservation | 0.635 | | | 0.424**±**0.218 | <0.0001 | |
| *Q. rubra* | Aboveground - Belowground | 0.227 | | | 0.404**±**0.208 | <0.0001 | |

**Supplementary Table S5** – Trait Correlation Matrix estimated using Bayesian Hierarchical Analysis. Traits are colored according to whether they are aboveground (green) or belowground (brown). Values in bold represent significant values determined by whether their 95% CI overlapped with zero.

| ***A. rubrum*** |  |  |  |  |  |  |
| --- | --- | --- | --- | --- | --- | --- |
|  |  |  |  |  |  |  |
| **Trait** | **SLA** | **SRL** | **RTD** | **RD** | **LfN** | **RtN** |
| **Specific Leaf Area (SLA)** | 1 | -0.264 | -0.027 | 0.203 | 0.006 | -0.277 |
| **Specific Root Length (SRL)** |  | 1 | **-0.511** | **-0.887** | 0.205 | **0.855** |
| **Root Tissue Density (RTD)** |  |  | 1 | 0.348 | 0.258 | -0.37 |
| **Root Diameter (RD)** |  |  |  | 1 | -0.311 | **-0.825** |
| **Leaf Nitrogen (LfN)** |  |  |  |  | 1 | 0.373 |
| **Root Nitrogen (RtN)** |  |  |  |  |  | 1 |
|  |  |  |  |  |  |  |
| ***A. saccharum*** |  |  |  |  |  |  |
|  |  |  |  |  |  |  |
| **Trait** | **SLA** | **SRL** | **RTD** | **RD** | **LfN** | **RtN** |
| **Specific Leaf Area (SLA)** | 1 | 0.21 | -0.107 | -0.181 | -0.229 | 0.24 |
| **Specific Root Length (SRL)** |  | 1 | **-0.851** | **-0.953** | **0.365** | **0.775** |
| **Root Tissue Density (RTD)** |  |  | 1 | **0.762** | **-0.334** | **-0.643** |
| **Root Diameter (RD)** |  |  |  | 1 | **-0.328** | **-0.816** |
| **Leaf Nitrogen (LfN)** |  |  |  |  | 1 | 0.171 |
| **Root Nitrogen (RtN)** |  |  |  |  |  | 1 |
|  |  |  |  |  |  |  |
| ***P. serotina*** |  |  |  |  |  |  |
|  |  |  |  |  |  |  |
| **Trait** | **SLA** | **SRL** | **RTD** | **RD** | **LfN** | **RtN** |
| **Specific Leaf Area (SLA)** | 1 | 0.075 | 0.283 | 0.068 | 0.125 | -0.134 |
| **Specific Root Length (SRL)** |  | 1 | **-0.436** | **-0.731** | 0.15 | 0.208 |
| **Root Tissue Density (RTD)** |  |  | 1 | **0.676** | 0.068 | -0.333 |
| **Root Diameter (RD)** |  |  |  | 1 | -0.221 | -0.559 |
| **Leaf Nitrogen (LfN)** |  |  |  |  | 1 | 0.272 |
| **Root Nitrogen (RtN)** |  |  |  |  |  | 1 |
|  |  |  |  |  |  |  |
| ***Q. rubra*** |  |  |  |  |  |  |
|  |  |  |  |  |  |  |
| **Trait** | **SLA** | **SRL** | **RTD** | **RD** | **LfN** | **RtN** |
| **Specific Leaf Area (SLA)** | 1 | 0.146 | -0.245 | **-0.362** | -0.038 | **0.332** |
| **Specific Root Length (SRL)** |  | 1 | **-0.726** | **-0.793** | 0.251 | **0.509** |
| **Root Tissue Density (RTD)** |  |  | 1 | **0.515** | -0.022 | -0.21 |
| **Root Diameter (RD)** |  |  |  | 1 | -0.178 | **-0.741** |
| **Leaf Nitrogen (LfN)** |  |  |  |  | 1 | **0.326** |
| **Root Nitrogen (RtN)** |  |  |  |  |  | 1 |

**Supplementary Table S6** – Bayesian hierarchical model results for the intercept and coefficients for light, total P, and total N for each species and Leaf N (LfN). Bolded values indicate significant values as 95% confidence interval (CI) did not overlap with zero, letters indicate values that differ from one another within a trait. Posterior Mean: mean value for the parameter.

| **Coefficient** | **Species** | **Trait** | **Posterior Mean** | **95%CI** | **Mycorrhizae** | **Sig from Other Species** |
| --- | --- | --- | --- | --- | --- | --- |
| Intercept | *A. rubrum* | LfN | 0.035 | ( -0.55, 0.69 ) | AM | a |
| Intercept | *A. saccharum* | LfN | -0.030 | ( -0.4 , 0.35 ) | AM | a |
| Intercept | *P. serotina* | LfN | 0.057 | ( -0.47 , 0.56 ) | AM | a |
| Intercept | *Q. rubra* | LfN | -0.025 | ( -0.32 , 0.27 ) | EM | a |
| **Light** | ***Q. rubra*** | **LfN** | **-0.484** | **( -0.77 , -0.2 )** | **EM** | **a** |
| Light | *A. rubrum* | LfN | -0.225 | ( -0.74 , 0.27 ) | AM | a |
| **Light** | ***A. saccharum*** | **LfN** | **-0.287** | **( -0.57 , -0.01 )** | **AM** | **a** |
| Light | *P. serotina* | LfN | -0.200 | ( -0.58 , 0.18 ) | AM | a |
| Total N | *A. rubrum* | LfN | 0.579 | ( -0.24 , 1.43 ) | AM | a |
| **Total N** | ***Q. rubra*** | **LfN** | **0.500** | **( 0.09 , 0.93 )** | **EM** | **a** |
| **Total N** | ***A. saccharum*** | **LfN** | **0.636** | **( 0.17 , 1.1 )** | **AM** | **a** |
| Total N | *P. serotina* | LfN | -0.286 | ( -0.94 , 0.39 ) | AM | a |
| Total P | *Q. rubra* | LfN | -0.363 | ( -0.81 , 0.08 ) | EM | a |
| Total P | *A. rubrum* | LfN | -0.468 | ( -1.39 , 0.4 ) | AM | a |
| **Total P** | ***A. saccharum*** | **LfN** | **-0.597** | **( -1.03 , -0.16 )** | **AM** | **a** |
| Total P | *P. serotina* | LfN | -0.324 | ( -0.98 , 0.36 ) | AM | a |

**Supplementary Table S7** – Bayesian hierarchical model results for the intercept and coefficients for light, total P, and total N for each species and Specific Leaf Area (SLA). Bolded values indicate significant values as 95% confidence interval (CI) did not overlap with zero, letters indicate values that differ from one another within a trait. Posterior Mean: mean value for the parameter.

| **Coefficient** | **Species** | **Trait** | **Posterior Mean** | **95%CI** | **Mycorrhizae** | **Sig from Other Species** |
| --- | --- | --- | --- | --- | --- | --- |
| Intercept | *A. rubrum* | SLA | -0.028 | ( -0.59 , 0.57 ) | AM | a |
| Intercept | *A. saccharum* | SLA | -0.010 | ( -0.4 , 0.41 ) | AM | a |
| Intercept | *P. serotina* | SLA | -0.086 | ( -0.56 , 0.41 ) | AM | a |
| Intercept | *Q. rubra* | SLA | 0.033 | ( -0.29 , 0.37 ) | EM | a |
| Light | *Q. rubra* | SLA | -0.253 | ( -0.57 , 0.06 ) | EM | a |
| Light | *A. rubrum* | SLA | 0.025 | ( -0.45 , 0.5 ) | AM | a |
| Light | *A. saccharum* | SLA | -0.004 | ( -0.38 , 0.36 ) | AM | a |
| Light | *P. serotina* | SLA | -0.142 | ( -0.52 , 0.23 ) | AM | a |
| Total N | *A. rubrum* | SLA | -0.512 | ( -1.24 , 0.23 ) | AM | a |
| Total N | *Q. rubra* | SLA | 0.219 | ( -0.25 , 0.68 ) | EM | a |
| Total N | *A. saccharum* | SLA | -0.149 | ( -0.66 , 0.38 ) | AM | a |
| Total N | *P. serotina* | SLA | 0.319 | ( -0.33 , 0.95 ) | AM | a |
| **Total P** | ***Q. rubra*** | **SLA** | **-0.504** | **( -0.98 , -0.02 )** | **EM** | **a** |
| Total P | *A. rubrum* | SLA | -0.137 | ( -0.97 , 0.67 ) | AM | a |
| Total P | *A. saccharum* | SLA | -0.121 | ( -0.65 , 0.44 ) | AM | a |
| Total P | *P. serotina* | SLA | -0.579 | ( -1.26 , 0.05 ) | AM | a |

**Supplementary Table S8** – Bayesian hierarchical model results for the intercept and coefficients for light, total P, and total N for each species and Root N (RtN). Bolded values indicate significant values as 95% confidence interval (CI) did not overlap with zero, letters indicate values that differ from one another within a trait. Posterior Mean: mean value for the parameter.

| **Coefficient** | **Species** | **Trait** | **Posterior Mean** | **95%CI** | **Mycorrhizae** | **Sig from Other Species** |
| --- | --- | --- | --- | --- | --- | --- |
| Intercept | *A. rubrum* | RtN | -0.079 | ( -0.67 , 0.45 ) | AM | a |
| Intercept | *A. saccharum* | RtN | 0.040 | ( -0.34 , 0.47 ) | AM | a |
| Intercept | *P. serotina* | RtN | -0.055 | ( -0.55 , 0.4 ) | AM | a |
| Intercept | *Q. rubra* | RtN | -0.034 | ( -0.34 , 0.26 ) | EM | a |
| Light | *Q. rubra* | RtN | -0.173 | ( -0.49 , 0.13 ) | EM | a |
| **Light** | ***A. rubrum*** | **RtN** | **-0.358** | **( -0.7 , -0.02 )** | **AM** | **a** |
| Light | *A. saccharum* | RtN | -0.181 | ( -0.5 , 0.13 ) | AM | a |
| Light | *P. serotina* | RtN | -0.121 | ( -0.39 , 0.15 ) | AM | a |
| Total N | *A. rubrum* | RtN | 0.151 | ( -0.65 , 0.75 ) | AM | a |
| **Total N** | ***Q. rubra*** | **RtN** | **0.495** | **( 0 , 0.95 )** | **EM** | **a** |
| **Total N** | ***A. saccharum*** | **RtN** | **0.554** | **( 0.04 , 1.02 )** | **AM** | **a** |
| **Total N** | ***P. serotina*** | **RtN** | **0.791** | **( 0.28 , 1.34 )** | **AM** | **a** |
| **Total P** | ***Q. rubra*** | **RtN** | **-0.729** | **( -1.2 , -0.22 )** | **EM** | **a** |
| Total P | *A. rubrum* | RtN | -0.554 | ( -1.28 , 0.21 ) | AM | a |
| Total P | *A. saccharum* | RtN | -0.268 | ( -0.7 , 0.16 ) | AM | a |
| **Total P** | ***P. serotina*** | **RtN** | **-0.904** | **( -1.46 , -0.39 )** | **AM** | **a** |

**Supplementary Table S9** – Bayesian hierarchical model results for the intercept and coefficients for light, total P, and total N for each species and Root Tissue Density (RTD). Bolded values indicate significant values as 95% confidence interval (CI) did not overlap with zero, letters indicate values that differ from one another within a trait. Posterior Mean: mean value for the parameter.

| **Coefficient** | **Species** | **Trait** | **Posterior Mean** | **95%CI** | **Mycorrhizae** | **Sig from Other Species** |
| --- | --- | --- | --- | --- | --- | --- |
| Intercept | *A. rubrum* | RTD | 0.112 | ( -0.51 , 0.76 ) | AM | a |
| Intercept | *A. saccharum* | RTD | -0.009 | ( -0.42 , 0.43 ) | AM | a |
| Intercept | *P. serotina* | RTD | 0.053 | ( -0.4 , 0.52 ) | AM | a |
| Intercept | *Q. rubra* | RTD | 0.062 | ( -0.22 , 0.35 ) | EM | a |
| Light | *Q. rubra* | RTD | 0.206 | ( -0.09 , 0.55 ) | EM | a |
| Light | *A. rubrum* | RTD | -0.013 | ( -0.43 , 0.41 ) | AM | a |
| Light | *A. saccharum* | RTD | 0.091 | ( -0.28 , 0.45 ) | AM | a |
| Light | *P. serotina* | RTD | 0.205 | ( -0.17 , 0.53 ) | AM | a |
| Total N | *A. rubrum* | RTD | 0.242 | ( -0.44 , 0.92 ) | AM | a |
| Total N | *Q. rubra* | RTD | -0.204 | ( -0.68 , 0.27 ) | EM | a |
| Total N | *A. saccharum* | RTD | -0.038 | ( -0.57 , 0.44 ) | AM | a |
| Total N | *P. serotina* | RTD | -0.373 | ( -0.91 , 0.26 ) | AM | a |
| **Total P** | ***Q. rubra*** | **RTD** | **0.712** | **( 0.2 , 1.17 )** | **EM** | **a** |
| Total P | *A. rubrum* | RTD | 0.153 | ( -0.51 , 0.97 ) | AM | a |
| Total P | *A. saccharum* | RTD | -0.220 | ( -0.65 , 0.3 ) | AM | a |
| **Total P** | ***P. serotina*** | **RTD** | **0.626** | **( 0.04 , 1.21 )** | **AM** | **a** |

**Supplementary Table S10** – Bayesian hierarchical model results for the intercept and coefficients for light, total P, and total N for each species and Root Diameter (RD). Bolded values indicate significant values as 95% confidence interval (CI) did not overlap with zero, letters indicate values that differ from one another within a trait. Posterior Mean: mean value for the parameter.

| **Coefficient** | **Species** | **Trait** | **Posterior Mean** | **95%CI** | **Mycorrhizae** | **Sig from Other Species** |
| --- | --- | --- | --- | --- | --- | --- |
| Intercept | *A. rubrum* | RD | -0.024 | ( -0.67 , 0.63 ) | AM | a |
| Intercept | *A. saccharum* | RD | 0.035 | ( -0.38 , 0.5 ) | AM | a |
| Intercept | *P. serotina* | RD | 0.255 | ( -0.2 , 0.82 ) | AM | a |
| Intercept | *Q. rubra* | RD | 0.027 | ( -0.29 , 0.32 ) | EM | a |
| **Light** | ***Q. rubra*** | **RD** | **0.468** | **( 0.17 , 0.83 )** | **EM** | **a** |
| Light | *A. rubrum* | RD | 0.070 | ( -0.43 , 0.57 ) | AM | a |
| Light | *A. saccharum* | RD | -0.008 | ( -0.4 , 0.37 ) | AM | a |
| Light | *P. serotina* | RD | -0.098 | ( -0.38 , 0.18 ) | AM | a |
| Total N | *A. rubrum* | RD | 0.263 | ( -0.47 , 1.1 ) | AM | a |
| Total N | *Q. rubra* | RD | 0.148 | ( -0.28 , 0.57 ) | EM | a |
| Total N | *A. saccharum* | RD | -0.122 | ( -0.68 , 0.47 ) | AM | a |
| Total N | *P. serotina* | RD | -0.280 | ( -0.87 , 0.27 ) | AM | a |
| Total P | *Q. rubra* | RD | 0.349 | ( -0.14 , 0.83 ) | EM | a |
| Total P | *A. rubrum* | RD | -0.031 | ( -0.82 , 0.74 ) | AM | a |
| Total P | *A. saccharum* | RD | -0.036 | ( -0.56 , 0.52 ) | AM | a |
| Total P | *P. serotina* | RD | 0.365 | ( -0.19 , 0.97 ) | AM | a |

**Supplementary Table S11** – Bayesian hierarchical model results for the intercept and coefficients for light, total P, and total N for each species and Specific Root Length (SRL). Bolded values indicate significant values as 95% confidence interval (CI) did not overlap with zero, letters indicate values that differ from one another within a trait. Posterior Mean: mean value for the parameter.

| **Coefficient** | **Species** | **Trait** | **Posterior Mean** | **95%CI** | **Mycorrhizae** | **Sig from Other Species** |
| --- | --- | --- | --- | --- | --- | --- |
| Intercept | *A. rubrum* | SRL | -0.080 | ( -0.59 , 0.44 ) | AM | a |
| Intercept | *A. saccharum* | SRL | 0.000 | ( -0.39 , 0.39 ) | AM | a |
| Intercept | *P. serotina* | SRL | -0.146 | ( -0.62 , 0.32 ) | AM | a |
| Intercept | *Q. rubra* | SRL | -0.020 | ( -0.36 , 0.33 ) | EM | a |
| Light | *Q. rubra* | SRL | -0.300 | ( -0.68 , 0.02 ) | EM | a |
| Light | *A. rubrum* | SRL | 0.014 | ( -0.45 , 0.45 ) | AM | a |
| Light | *A. saccharum* | SRL | -0.097 | ( -0.45 , 0.26 ) | AM | a |
| Light | *P. serotina* | SRL | -0.260 | ( -0.6 , 0.13 ) | AM | a |
| Total N | *A. rubrum* | SRL | -0.382 | ( -1.14 , 0.21 ) | AM | a |
| Total N | *Q. rubra* | SRL | 0.204 | ( -0.23 , 0.63 ) | EM | a |
| Total N | *A. saccharum* | SRL | -0.014 | ( -0.6 , 0.5 ) | AM | a |
| Total N | *P. serotina* | SRL | -0.013 | ( -0.63 , 0.65 ) | AM | a |
| **Total P** | ***Q. rubra*** | **SRL** | **-0.574** | **( -1.03 , -0.11 )** | **EM** | **a** |
| Total P | *A. rubrum* | SRL | 0.007 | ( -0.64 , 0.81 ) | AM | a |
| Total P | *A. saccharum* | SRL | 0.114 | ( -0.41 , 0.59 ) | AM | a |
| Total P | *P. serotina* | SRL | -0.236 | ( -0.99 , 0.43 ) | AM | a |

**Supplementary Table S12** – Bayesian hierarchical model results for the coefficients of site random effects for each species, trait, site combination. Bolded values indicate significant values as 95% credible interval (CI) did not overlap with zero, Posterior Mean: mean value for the parameter.

| **Species** | **Site** | **Location** | **Trait** | **Latitude** | **Posterior Mean** | **95% CI** |
| --- | --- | --- | --- | --- | --- | --- |
| *A. rubrum* | Aspen | North 1 | SLA | North | 0.294 | (-0.69,1.25) |
| *A. rubrum* | Balsam Fir | North 2 | SLA | North | -0.006 | (-1.24,1.17) |
| *A. rubrum* | ESGR | South 1 | SLA | South | 0.056 | (-0.96,1.22) |
| *A. rubrum* | Northern Hardwoods | North 3 | SLA | North | 0.297 | (-0.67,1.44) |
| *A. rubrum* | Pine-Aspen | North 4 | SLA | North | -0.735 | (-1.7,0.13) |
| *A. rubrum* | Radrick Forest | South 2 | SLA | South | -0.063 | (-0.99,0.84) |
| *A. rubrum* | Saginaw Forest | South 3 | SLA | South | 0.463 | (-0.37,1.37) |
| *A. rubrum* | Stinchfield Woods | South 4 | SLA | South | -0.216 | (-1.15,0.68) |
| *A. rubrum* | Aspen | North 1 | LfN | North | -0.043 | (-1.07,0.95) |
| *A. rubrum* | Balsam Fir | North 2 | LfN | North | 0.015 | (-1.12,1.27) |
| *A. rubrum* | ESGR | South 1 | LfN | South | 0.136 | (-1.06,1.2) |
| *A. rubrum* | Northern Hardwoods | North 3 | LfN | North | -0.002 | (-1.08,0.97) |
| *A. rubrum* | Pine-Aspen | North 4 | LfN | North | -0.070 | (-1,0.82) |
| *A. rubrum* | Radrick Forest | South 2 | LfN | South | 0.262 | (-0.76,1.3) |
| *A. rubrum* | Saginaw Forest | South 3 | LfN | South | -0.127 | (-1.12,0.91) |
| *A. rubrum* | Stinchfield Woods | South 4 | LfN | South | -0.324 | (-1.39,0.59) |
| *A. rubrum* | Aspen | North 1 | RtN | North | 0.673 | (-0.17,1.52) |
| *A. rubrum* | Balsam Fir | North 2 | RtN | North | -0.027 | (-1.22,1.17) |
| *A. rubrum* | ESGR | South 1 | RtN | South | -0.185 | (-1.48,0.87) |
| *A. rubrum* | Northern Hardwoods | North 3 | RtN | North | -0.685 | (-1.61,0.13) |
| *A. rubrum* | Pine-Aspen | North 4 | RtN | North | -0.036 | (-0.84,0.71) |
| *A. rubrum* | Radrick Forest | South 2 | RtN | South | 0.379 | (-0.47,1.32) |
| *A. rubrum* | Saginaw Forest | South 3 | RtN | South | 0.193 | (-0.56,1.2) |
| *A. rubrum* | Stinchfield Woods | South 4 | RtN | South | -0.211 | (-1.07,0.62) |
| *A. rubrum* | Aspen | North 1 | RTD | North | -0.170 | (-1.23,0.75) |
| *A. rubrum* | Balsam Fir | North 2 | RTD | North | -0.014 | (-1.22,1.16) |
| *A. rubrum* | ESGR | South 1 | RTD | South | -0.303 | (-1.43,0.83) |
| *A. rubrum* | Northern Hardwoods | North 3 | RTD | North | -0.074 | (-0.94,0.95) |
| *A. rubrum* | Pine-Aspen | North 4 | RTD | North | 0.113 | (-0.79,1.23) |
| *A. rubrum* | Radrick Forest | South 2 | RTD | South | 0.191 | (-0.67,1.14) |
| *A. rubrum* | Saginaw Forest | South 3 | RTD | South | -0.289 | (-1.11,0.53) |
| *A. rubrum* | Stinchfield Woods | South 4 | RTD | South | 0.592 | (-0.17,1.47) |
| *A. rubrum* | Aspen | North 1 | SRL | North | 0.181 | (-0.77,1.08) |
| *A. rubrum* | Balsam Fir | North 2 | SRL | North | -0.006 | (-1.2,1.16) |
| *A. rubrum* | ESGR | South 1 | SRL | South | 0.003 | (-0.84,0.92) |
| *A. rubrum* | Northern Hardwoods | North 3 | SRL | North | -0.041 | (-0.97,0.87) |
| *A. rubrum* | Pine-Aspen | North 4 | SRL | North | -0.223 | (-0.95,0.48) |
| *A. rubrum* | Radrick Forest | South 2 | SRL | South | -0.173 | (-1.05,0.73) |
| *A. rubrum* | Saginaw Forest | South 3 | SRL | South | 0.370 | (-0.49,1.31) |
| *A. rubrum* | Stinchfield Woods | South 4 | SRL | South | -0.190 | (-1.06,0.65) |
| *A. rubrum* | Aspen | North 1 | RD | North | 0.057 | (-1.06,1.1) |
| *A. rubrum* | Balsam Fir | North 2 | RD | North | -0.003 | (-1.18,1.23) |
| *A. rubrum* | ESGR | South 1 | RD | South | -0.262 | (-1.26,0.82) |
| *A. rubrum* | Northern Hardwoods | North 3 | RD | North | 0.094 | (-0.85,1.16) |
| *A. rubrum* | Pine-Aspen | North 4 | RD | North | -0.083 | (-1.02,0.8) |
| *A. rubrum* | Radrick Forest | South 2 | RD | South | -0.389 | (-1.46,0.63) |
| *A. rubrum* | Saginaw Forest | South 3 | RD | South | 0.140 | (-0.89,1.1) |
| *A. rubrum* | Stinchfield Woods | South 4 | RD | South | 0.676 | (-0.28,1.77) |
| *A. saccharum* | Aspen | North 1 | SLA | North | 0.081 | (-0.45,0.65) |
| *A. saccharum* | Balsam Fir | North 2 | SLA | North | -0.002 | (-0.63,0.61) |
| *A. saccharum* | ESGR | South 1 | SLA | South | -0.142 | (-0.81,0.38) |
| *A. saccharum* | Northern Hardwoods | North 3 | SLA | North | -0.067 | (-0.61,0.46) |
| *A. saccharum* | Pine-Aspen | North 4 | SLA | North | 0.008 | (-0.62,0.61) |
| *A. saccharum* | Radrick Forest | South 2 | SLA | South | 0.080 | (-0.45,0.62) |
| *A. saccharum* | Saginaw Forest | South 3 | SLA | South | -0.184 | (-0.75,0.32) |
| *A. saccharum* | Stinchfield Woods | South 4 | SLA | South | 0.247 | (-0.21,0.86) |
| *A. saccharum* | Aspen | North 1 | LfN | North | -0.010 | (-0.5,0.49) |
| *A. saccharum* | Balsam Fir | North 2 | LfN | North | -0.004 | (-0.63,0.59) |
| *A. saccharum* | ESGR | South 1 | LfN | South | -0.036 | (-0.58,0.52) |
| *A. saccharum* | Northern Hardwoods | North 3 | LfN | North | -0.032 | (-0.56,0.49) |
| *A. saccharum* | Pine-Aspen | North 4 | LfN | North | 0.001 | (-0.58,0.59) |
| *A. saccharum* | Radrick Forest | South 2 | LfN | South | 0.137 | (-0.3,0.67) |
| *A. saccharum* | Saginaw Forest | South 3 | LfN | South | -0.177 | (-0.71,0.28) |
| *A. saccharum* | Stinchfield Woods | South 4 | LfN | South | 0.078 | (-0.39,0.59) |
| *A. saccharum* | Aspen | North 1 | RtN | North | -0.043 | (-0.54,0.47) |
| *A. saccharum* | Balsam Fir | North 2 | RtN | North | 0.010 | (-0.61,0.61) |
| *A. saccharum* | ESGR | South 1 | RtN | South | 0.141 | (-0.39,0.74) |
| *A. saccharum* | Northern Hardwoods | North 3 | RtN | North | -0.012 | (-0.56,0.43) |
| *A. saccharum* | Pine-Aspen | North 4 | RtN | North | -0.007 | (-0.61,0.56) |
| *A. saccharum* | Radrick Forest | South 2 | RtN | South | 0.233 | (-0.24,0.7) |
| *A. saccharum* | Saginaw Forest | South 3 | RtN | South | -0.057 | (-0.57,0.4) |
| *A. saccharum* | Stinchfield Woods | South 4 | RtN | South | -0.331 | (-0.88,0.09) |
| *A. saccharum* | Aspen | North 1 | RTD | North | -0.092 | (-0.59,0.4) |
| *A. saccharum* | Balsam Fir | North 2 | RTD | North | 0.010 | (-0.57,0.62) |
| *A. saccharum* | ESGR | South 1 | RTD | South | 0.051 | (-0.54,0.73) |
| *A. saccharum* | Northern Hardwoods | North 3 | RTD | North | 0.090 | (-0.33,0.49) |
| *A. saccharum* | Pine-Aspen | North 4 | RTD | North | -0.009 | (-0.6,0.58) |
| *A. saccharum* | Radrick Forest | South 2 | RTD | South | 0.026 | (-0.47,0.44) |
| *A. saccharum* | Saginaw Forest | South 3 | RTD | South | -0.141 | (-0.59,0.3) |
| *A. saccharum* | Stinchfield Woods | South 4 | RTD | South | 0.046 | (-0.45,0.53) |
| *A. saccharum* | Aspen | North 1 | SRL | North | -0.029 | (-0.55,0.47) |
| *A. saccharum* | Balsam Fir | North 2 | SRL | North | -0.010 | (-0.64,0.59) |
| *A. saccharum* | ESGR | South 1 | SRL | South | -0.004 | (-0.51,0.5) |
| *A. saccharum* | Northern Hardwoods | North 3 | SRL | North | -0.045 | (-0.5,0.44) |
| *A. saccharum* | Pine-Aspen | North 4 | SRL | North | -0.006 | (-0.62,0.61) |
| *A. saccharum* | Radrick Forest | South 2 | SRL | South | 0.207 | (-0.25,0.8) |
| *A. saccharum* | Saginaw Forest | South 3 | SRL | South | -0.156 | (-0.68,0.37) |
| *A. saccharum* | Stinchfield Woods | South 4 | SRL | South | 0.048 | (-0.35,0.54) |
| *A. saccharum* | Aspen | North 1 | RD | North | 0.058 | (-0.61,0.59) |
| *A. saccharum* | Balsam Fir | North 2 | RD | North | 0.009 | (-0.62,0.61) |
| *A. saccharum* | ESGR | South 1 | RD | South | -0.046 | (-0.58,0.48) |
| *A. saccharum* | Northern Hardwoods | North 3 | RD | North | -0.115 | (-0.66,0.35) |
| *A. saccharum* | Pine-Aspen | North 4 | RD | North | -0.004 | (-0.6,0.61) |
| *A. saccharum* | Radrick Forest | South 2 | RD | South | 0.008 | (-0.52,0.5) |
| *A.saccharum* | Saginaw Forest | South 3 | RD | South | 0.057 | (-0.44,0.64) |
| *A.saccharum* | Stinchfield Woods | South 4 | RD | South | -0.028 | (-0.52,0.44) |
| *P. serotina* | Aspen | North 1 | SLA | North | -0.090 | (-1,0.72) |
| *P. serotina* | Balsam Fir | North 2 | SLA | North | 0.002 | (-1.05,1.06) |
| *P. serotina* | ESGR | South 1 | SLA | South | 0.055 | (-0.94,1.04) |
| *P. serotina* | Northern Hardwoods | North 3 | SLA | North | -0.020 | (-0.88,0.86) |
| *P. serotina* | Pine-Aspen | North 4 | SLA | North | 0.153 | (-0.58,0.89) |
| *P. serotina* | Radrick Forest | South 2 | SLA | South | 0.503 | (-0.23,1.34) |
| *P. serotina* | Saginaw Forest | South 3 | SLA | South | -0.412 | (-1.2,0.35) |
| *P. serotina* | Stinchfield Woods | South 4 | SLA | South | -0.105 | (-0.87,0.67) |
| *P. serotina* | Aspen | North 1 | LfN | North | 0.034 | (-0.86,0.96) |
| *P. serotina* | Balsam Fir | North 2 | LfN | North | 0.022 | (-0.99,1.06) |
| *P. serotina* | ESGR | South 1 | LfN | South | 0.017 | (-0.82,0.95) |
| *P. serotina* | Northern Hardwoods | North 3 | LfN | North | 0.243 | (-0.62,1.2) |
| *P. serotina* | Pine-Aspen | North 4 | LfN | North | -0.258 | (-1.03,0.52) |
| *P. serotina* | Radrick Forest | South 2 | LfN | South | 0.168 | (-0.6,0.94) |
| *P. serotina* | Saginaw Forest | South 3 | LfN | South | -0.159 | (-0.99,0.65) |
| *P. serotina* | Stinchfield Woods | South 4 | LfN | South | -0.043 | (-0.81,0.68) |
| *P. serotina* | Aspen | North 1 | RtN | North | 0.073 | (-0.68,0.89) |
| *P. serotina* | Balsam Fir | North 2 | RtN | North | 0.013 | (-1.05,1.01) |
| *P. serotina* | ESGR | South 1 | RtN | South | 0.151 | (-0.78,1.14) |
| *P. serotina* | Northern Hardwoods | North 3 | RtN | North | -0.100 | (-0.9,0.68) |
| *P. serotina* | Pine-Aspen | North 4 | RtN | North | -0.056 | (-0.72,0.62) |
| *P. serotina* | Radrick Forest | South 2 | RtN | South | 0.245 | (-0.4,0.96) |
| *P. serotina* | Saginaw Forest | South 3 | RtN | South | 0.154 | (-0.53,0.9) |
| *P. serotina* | Stinchfield Woods | South 4 | RtN | South | -0.305 | (-1.1,0.43) |
| *P. serotina* | Aspen | North 1 | RTD | North | 0.337 | (-0.44,1.18) |
| *P. serotina* | Balsam Fir | North 2 | RTD | North | -0.013 | (-1.05,1.03) |
| *P. serotina* | ESGR | South 1 | RTD | South | -0.196 | (-1.21,0.64) |
| *P. serotina* | Northern Hardwoods | North 3 | RTD | North | -0.297 | (-1.12,0.52) |
| *P. serotina* | Pine-Aspen | North 4 | RTD | North | -0.163 | (-0.95,0.52) |
| *P. serotina* | Radrick Forest | South 2 | RTD | South | 0.070 | (-0.62,0.73) |
| *P. serotina* | Saginaw Forest | South 3 | RTD | South | 0.059 | (-0.67,0.74) |
| *P. serotina* | Stinchfield Woods | South 4 | RTD | South | 0.113 | (-0.56,0.89) |
| *P. serotina* | Aspen | North 1 | SRL | North | -0.072 | (-0.91,0.76) |
| *P. serotina* | Balsam Fir | North 2 | SRL | North | 0.012 | (-1.04,0.98) |
| *P. serotina* | ESGR | South 1 | SRL | South | -0.001 | (-0.94,0.94) |
| *P. serotina* | Northern Hardwoods | North 3 | SRL | North | -0.266 | (-1.13,0.59) |
| *P. serotina* | Pine-Aspen | North 4 | SRL | North | 0.548 | (-0.23,1.36) |
| *P. serotina* | Radrick Forest | South 2 | SRL | South | -0.181 | (-0.87,0.53) |
| *P. serotina* | Saginaw Forest | South 3 | SRL | South | 0.009 | (-0.76,0.74) |
| *P. serotina* | Stinchfield Woods | South 4 | SRL | South | -0.019 | (-0.76,0.67) |
| *P. serotina* | Aspen | North 1 | RD | North | 0.419 | (-0.43,1.28) |
| *P. serotina* | Balsam Fir | North 2 | RD | North | -0.007 | (-1.04,1.04) |
| *P. serotina* | ESGR | South 1 | RD | South | -0.291 | (-1.34,0.66) |
| *P. serotina* | Northern Hardwoods | North 3 | RD | North | 0.375 | (-0.41,1.21) |
| *P. serotina* | Pine-Aspen | North 4 | RD | North | -1.023 | (-1.78,-0.28) |
| *P. serotina* | Radrick Forest | South 2 | RD | South | 0.261 | (-0.43,0.92) |
| *P. serotina* | Saginaw Forest | South 3 | RD | South | -0.001 | (-0.72,0.7) |
| *P. serotina* | Stinchfield Woods | South 4 | RD | South | 0.217 | (-0.49,0.95) |
| *Q. rubra* | Aspen | North 1 | SLA | North | 0.028 | (-0.38,0.46) |
| *Q. rubra* | Balsam Fir | North 2 | SLA | North | -0.084 | (-0.55,0.29) |
| *Q. rubra* | ESGR | South 1 | SLA | South | -0.030 | (-0.54,0.39) |
| *Q. rubra* | Northern Hardwoods | North 3 | SLA | North | -0.015 | (-0.42,0.37) |
| *Q. rubra* | Pine-Aspen | North 4 | SLA | North | 0.062 | (-0.33,0.56) |
| *Q. rubra* | Radrick Forest | South 2 | SLA | South | 0.150 | (-0.2,0.64) |
| *Q. rubra* | Saginaw Forest | South 3 | SLA | South | -0.119 | (-0.61,0.24) |
| *Q. rubra* | Stinchfield Woods | South 4 | SLA | South | -0.027 | (-0.43,0.38) |
| *Q. rubra* | Aspen | North 1 | LfN | North | -0.025 | (-0.44,0.34) |
| *Q. rubra* | Balsam Fir | North 2 | LfN | North | 0.079 | (-0.28,0.5) |
| *Q. rubra* | ESGR | South 1 | LfN | South | 0.045 | (-0.37,0.53) |
| *Q. rubra* | Northern Hardwoods | North 3 | LfN | North | -0.121 | (-0.58,0.22) |
| *Q. rubra* | Pine-Aspen | North 4 | LfN | North | 0.055 | (-0.32,0.47) |
| *Q. rubra* | Radrick Forest | South 2 | LfN | South | -0.011 | (-0.45,0.4) |
| *Q. rubra* | Saginaw Forest | South 3 | LfN | South | 0.050 | (-0.35,0.5) |
| *Q. rubra* | Stinchfield Woods | South 4 | LfN | South | -0.088 | (-0.54,0.26) |
| *Q. rubra* | Aspen | North 1 | RtN | North | -0.110 | (-0.52,0.25) |
| *Q. rubra* | Balsam Fir | North 2 | RtN | North | -0.068 | (-0.48,0.29) |
| *Q. rubra* | ESGR | South 1 | RtN | South | 0.049 | (-0.4,0.52) |
| *Q. rubra* | Northern Hardwoods | North 3 | RtN | North | 0.153 | (-0.2,0.63) |
| *Q. rubra* | Pine-Aspen | North 4 | RtN | North | 0.033 | (-0.35,0.43) |
| *Q. rubra* | Radrick Forest | South 2 | RtN | South | 0.062 | (-0.29,0.47) |
| *Q. rubra* | Saginaw Forest | South 3 | RtN | South | -0.108 | (-0.57,0.27) |
| *Q. rubra* | Stinchfield Woods | South 4 | RtN | South | -0.031 | (-0.42,0.36) |
| *Q. rubra* | Aspen | North 1 | RTD | North | -0.086 | (-0.49,0.26) |
| *Q. rubra* | Balsam Fir | North 2 | RTD | North | 0.012 | (-0.34,0.41) |
| *Q. rubra* | ESGR | South 1 | RTD | South | 0.077 | (-0.29,0.51) |
| *Q. rubra* | Northern Hardwoods | North 3 | RTD | North | -0.002 | (-0.38,0.33) |
| *Q. rubra* | Pine-Aspen | North 4 | RTD | North | 0.071 | (-0.29,0.43) |
| *Q. rubra* | Radrick Forest | South 2 | RTD | South | 0.052 | (-0.28,0.44) |
| *Q. rubra* | Saginaw Forest | South 3 | RTD | South | -0.044 | (-0.48,0.34) |
| *Q. rubra* | Stinchfield Woods | South 4 | RTD | South | -0.088 | (-0.44,0.27) |
| *Q. rubra* | Aspen | North 1 | SRL | North | 0.080 | (-0.28,0.59) |
| *Q. rubra* | Balsam Fir | North 2 | SRL | North | -0.075 | (-0.5,0.24) |
| *Q. rubra* | ESGR | South 1 | SRL | South | 0.043 | (-0.44,0.5) |
| *Q. rubra* | Northern Hardwoods | North 3 | SRL | North | 0.056 | (-0.31,0.48) |
| *Q. rubra* | Pine-Aspen | North 4 | SRL | North | -0.044 | (-0.44,0.3) |
| *Q. rubra* | Radrick Forest | South 2 | SRL | South | -0.127 | (-0.61,0.19) |
| *Q. rubra* | Saginaw Forest | South 3 | SRL | South | 0.212 | (-0.12,0.72) |
| *Q. rubra* | Stinchfield Woods | South 4 | SRL | South | -0.130 | (-0.57,0.2) |
| *Q. rubra* | Aspen | North 1 | RD | North | -0.125 | (-0.62,0.22) |
| *Q. rubra* | Balsam Fir | North 2 | RD | North | 0.128 | (-0.21,0.61) |
| *Q. rubra* | ESGR | South 1 | RD | South | 0.079 | (-0.35,0.54) |
| *Q. rubra* | Northern Hardwoods | North 3 | RD | North | 0.023 | (-0.37,0.42) |
| *Q. rubra* | Pine-Aspen | North 4 | RD | North | -0.022 | (-0.44,0.4) |
| *Q. rubra* | Radrick Forest | South 2 | RD | South | 0.097 | (-0.24,0.54) |
| *Q. rubra* | Saginaw Forest | South 3 | RD | South | -0.061 | (-0.56,0.3) |
| *Q. rubra* | Stinchfield Woods | South 4 | RD | South | -0.068 | (-0.45,0.3) |

**Supplementary Figure S1 –** Boxplots showing mean trait values and distribution of data for the two aboveground traits measured for each of the four species. Each point represents the trait value of a single individual. Letters indicate significant differences between species as determined by two-way ANOVA.


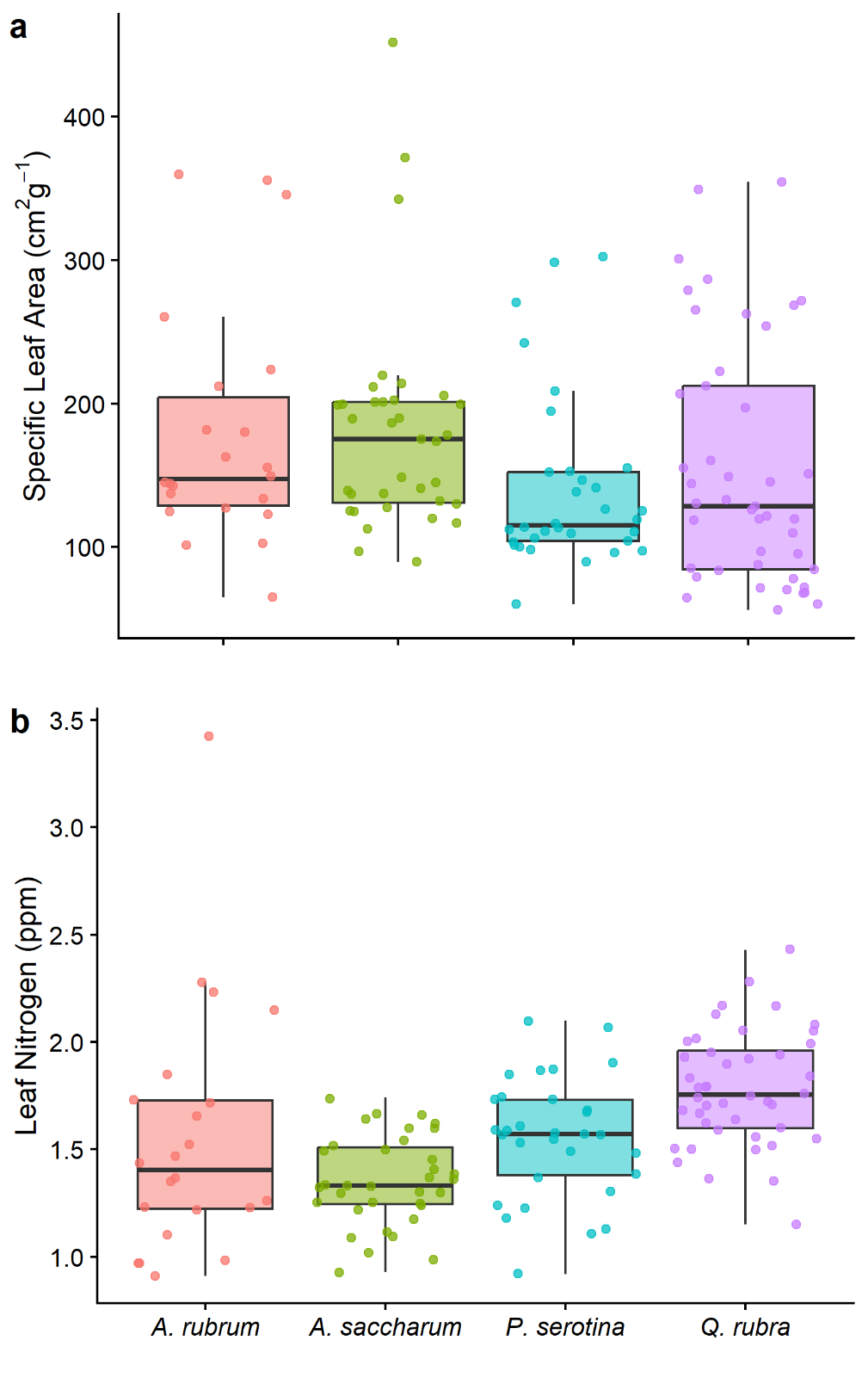


**Supplementary Figure S2 –** Boxplots showing mean trait values and distribution of data for the four belowground traits measured for each of the four species. Each point represents the trait value of a single individual. Letters indicate significant differences between species as determined by two-way ANOVA.

**
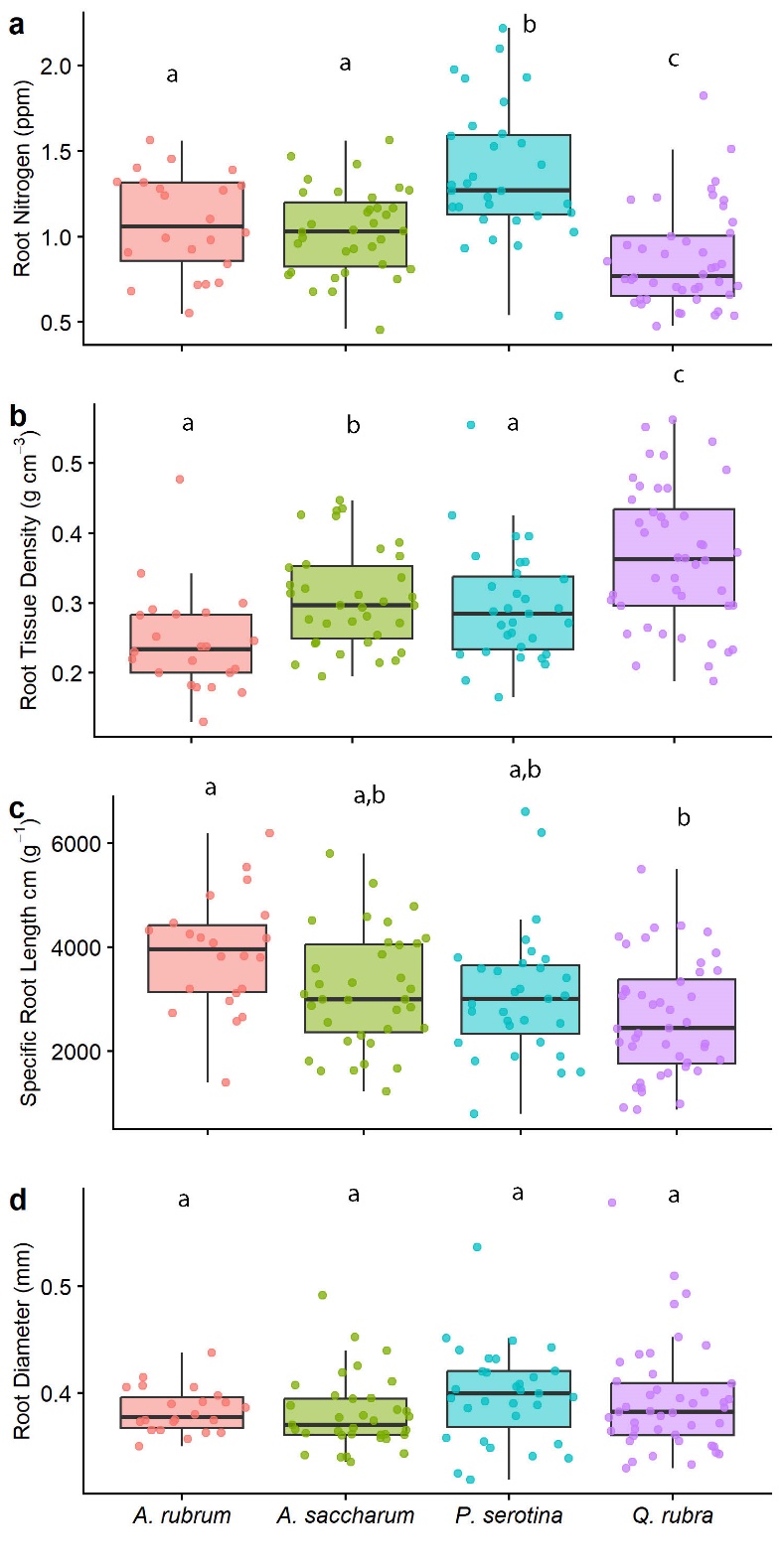
**

**Model Code**

model{

for(i in 1:22){ #acru

trait.acru[i,1:6]~dmnorm(T.acru[i,],S.acru[,])

for(t in 1:6){

T.acru[i,t]<-alpha1[t]+beta1[t]*totalN.acru[i]+ beta2[t]*totalP.acru[i]+ beta3[t]*light.acru[i]+ SRE.acru[site.acru[i]]

}

}

for(i in 1:34){ #acsa

trait.acsa[i,1:6]~dmnorm(T.acsa[i,],S.acsa[,])

for(t in 1:6){

T.acsa[i,t]<-alpha2[t]+beta4[t]*totalN.acsa[i]+ beta5[t]*totalP.acsa[i]+ beta6[t]*light.acsa[i]+ SRE.acsa[site.acsa[i]]

}

}

for(i in 1:32){ #prse

trait.prse[i,1:6]~dmnorm(T.prse[i,],S.prse[,])

for(t in 1:6){

T.prse[i,t]<-alpha3[t]+beta7[t]*totalN.prse[i]+ beta8[t]*totalP.prse[i]+ beta9[t]*light.prse[i]+ SRE.prse[site.prse[i]]

}

}

for(i in 1:43){ #quru

trait.quru[i,1:6]~dmnorm(T.quru[i,],S.quru[,])

for(t in 1:6){

T.quru[i,t]<-alpha4[t]+beta10[t]*totalN.quru[i]+ beta11[t]*totalP.quru[i]+ beta12[t]*light.quru[i]+ SRE.quru[site.quru[i]]

}

}

for( t in 1:6){

alpha1[t]~dnorm(0,0.001)

alpha2[t]~dnorm(0,0.001)

alpha3[t]~dnorm(0,0.001)

alpha4[t]~dnorm(0,0.001)

beta1[t]~dnorm(0,0.001)

beta2[t]~dnorm(0,0.001)

beta3[t]~dnorm(0,0.001)

beta4[t]~dnorm(0,0.001)

beta5[t]~dnorm(0,0.001)

beta6[t]~dnorm(0,0.001)

beta7[t]~dnorm(0,0.001)

beta8[t]~dnorm(0,0.001)

beta9[t]~dnorm(0,0.001)

beta10[t]~dnorm(0,0.001)

beta11[t]~dnorm(0,0.001)

beta12[t]~dnorm(0,0.001)

}

for( s in 1:8){

SRE.acru[s]~dnorm(0,tau[1])

SRE.acsa[s]~dnorm(0,tau[2])

SRE.prse[s]~dnorm(0,tau[3])

SRE.quru[s]~dnorm(0,tau[4])

}

for(i in 1:4){

tau[i]<-1/variance[i]

variance[i]~dunif(0,100)

}

S.acru[1:6,1:6]~dwish(R.acru[,],6) #add R as data, 6x6 matrix all zeros except the diagonal (=0.001)

S.acsa[1:6,1:6]~dwish(R.acsa[,],6)

S.prse[1:6,1:6]~dwish(R.prse[,],6)

S.quru[1:6,1:6]~dwish(R.quru[,],6)

#Convert S to sd and correlation:

Var.acru[1:6,1:6]<-inverse(S.acru[,])

for ( t in 1:6 ) { Sigma.acru[t] <- sqrt(Var.acru[t,t]) }

for ( t1.acru in 1:6 ) { for ( t2.acru in 1:6 ) {

Rho.acru[t1.acru,t2.acru] <- ( Var.acru[t1.acru,t2.acru]

/ (Sigma.acru[t1.acru]*Sigma.acru[t2.acru]) )

} }

Var.acsa[1:6,1:6]<-inverse(S.acsa[,])

for ( t in 1:6 ) { Sigma.acsa[t] <- sqrt(Var.acsa[t,t]) }

for ( t1.acsa in 1:6 ) { for ( t2.acsa in 1:6 ) {

Rho.acsa[t1.acsa,t2.acsa] <- ( Var.acsa[t1.acsa,t2.acsa]

/ (Sigma.acsa[t1.acsa]*Sigma.acsa[t2.acsa]) )

} }

Var.prse[1:6,1:6]<-inverse(S.prse[,])

for ( t in 1:6 ) { Sigma.prse[t] <- sqrt(Var.prse[t,t]) }

for ( t1.prse in 1:6 ) { for ( t2.prse in 1:6 ) {

Rho.prse[t1.prse,t2.prse] <- ( Var.prse[t1.prse,t2.prse]

/ (Sigma.prse[t1.prse]*Sigma.prse[t2.prse]) )

} }

Var.quru[1:6,1:6]<-inverse(S.quru[,])

for ( t in 1:6 ) { Sigma.quru[t] <- sqrt(Var.quru[t,t]) }

for ( t1.quru in 1:6 ) { for ( t2.quru in 1:6 ) {

Rho.quru[t1.quru,t2.quru] <- ( Var.quru[t1.quru,t2.quru]

/ (Sigma.quru[t1.quru]*Sigma.quru[t2.quru]) )

} }

}
